# Transcriptome dynamics associated with resistance and susceptibility against fusarium head blight in four wheat genotypes

**DOI:** 10.1186/s12864-018-5012-3

**Published:** 2018-08-29

**Authors:** Youlian Pan, Ziying Liu, Hélène Rocheleau, François Fauteux, Yunli Wang, Curt McCartney, Thérèse Ouellet

**Affiliations:** 1 Digital Technologies Research Centre, NRC, 1200 Montreal Road, Ottawa, ON K1A 0R6 Canada; 2 Ottawa Research and Development Centre, AAFC, 960 Carling Ave, Ottawa, ON K1A 0C6 Canada; 3 Morden Research and Development Centre, AAFC, 101 Route 100, Morden, MB R6M 1Y5 Canada

**Keywords:** Fusarium head blight, Differentially expressed genes, RNA-seq, *Triticum aestivum*, *Fusarium graminearum*, Pathogenesis, Plant defense

## Abstract

**Background:**

Fusarium head blight (FHB) of wheat in North America is caused mostly by the fungal pathogen *Fusarium graminearum* (*Fg*). Upon exposure to *Fg*, wheat initiates a series of cellular responses involving massive transcriptional reprogramming. In this study, we analyzed transcriptomics data of four wheat genotypes (Nyubai, Wuhan 1, HC374, and Shaw), at 2 and 4 days post inoculation (dpi) with *Fg*, using RNA-seq technology.

**Results:**

A total of 37,772 differentially expressed genes (DEGs) were identified, 28,961 from wheat and 8811 from the pathogen. The susceptible genotype Shaw exhibited the highest number of host and pathogen DEGs, including 2270 DEGs associating with FHB susceptibility. Protein serine/threonine kinases and *LRR-RK* were associated with susceptibility at 2 dpi, while several ethylene-responsive, *WRKY, Myb, bZIP* and *NAC*-domain containing transcription factors were associated with susceptibility at 4 dpi. In the three resistant genotypes, 220 DEGs were associated with resistance. Glutathione S-transferase (*GST*), membrane proteins and distinct *LRR-RKs* were associated with FHB resistance across the three genotypes. Genes with unique, high up-regulation by *Fg* in Wuhan 1 were mostly transiently expressed at 2 dpi, while many defense-associated genes were up-regulated at both 2 and 4 dpi in Nyubai; the majority of unique genes up-regulated in HC374 were detected at 4 dpi only. In the pathogen, most genes showed increased expression between 2 and 4 dpi in all genotypes, with stronger levels in the susceptible host; however two pectate lyases and a hydrolase were expressed higher at 2 dpi, and acetyltransferase activity was highly enriched at 4 dpi.

**Conclusions:**

There was an early up-regulation of *LRR-RKs,* different between susceptible and resistant genotypes; subsequently, distinct sets of genes associated with defense response were up-regulated. Differences in expression profiles among the resistant genotypes indicate genotype-specific defense mechanisms. This study also shows a greater resemblance in transcriptomics of HC374 to Nyubai, consistent with their sharing of two FHB resistance QTLs on 3BS and 5AS, compared to Wuhan 1 which carries one QTL on 2DL in common with HC374.

**Electronic supplementary material:**

The online version of this article (10.1186/s12864-018-5012-3) contains supplementary material, which is available to authorized users.

## Background

Wheat yield is severely limited by diseases caused by microbial pathogens. One of the prevalent wheat diseases, fusarium head blight (FHB), is caused by ascomycetous fungi of the genus *Fusarium*. The most common *Fusarium* species causing FHB in North America is *F. graminearum* [1]. *Fusarium graminearum* (*Fg*) produces deoxynivalenol (DON, also known as vomitoxin), the most prevalent trichothecene in Canadian wheat. Mycotoxin-contaminated grain is sold at lower prices or is completely rejected. Wheat resistance to FHB is categorized into five types: resistance to initial infection (type I), resistance to spread (type II), resistance to DON accumulation (type III), resistance to kernel infection (type IV), and tolerance (type V) [2].

Several FHB-resistant wheat cultivars have been identified and a large number of quantitative trait loci (QTLs) conferring resistance to FHB in wheat have been discovered. At least 22 chromosomal regions have been identified as contributing consistently to FHB resistance in multiple studies (reviewed in [3]). One of the most effective and best characterized sources of resistance against FHB is the Chinese cultivar Sumai 3 and its derivatives. These harbor a major QTL for type II resistance (up to 20–25% reduction of disease severity), named *Fhb1*, that has been mapped to chromosome 3BS as well as a minor QTL associated with type 1 resistance on chromosome 5AS, *Qfhs.ifa-5A*. QTLs in the same chromosomal regions of 3BS and 5AS have been detected in a range of FHB-resistant material, including the genotype Nyubai [4]. A minor QTL associated with type II resistance was also identified by the same authors in the Chinese genotype Wuhan 1 on chromosome 2DL. They also identified 3 QTLs (2DL, 3BS, and 5A) in the double haploid progeny HC374 after crossing Nyubai with Wuhan 1.

As result of exposure to pathogenic microorganisms, such as *Fg*, plants have evolved intricate mechanisms to recognize and defend themselves against potential infection. One of these responses is the down-regulation of photosynthesis and other processes associated with primary metabolisms that are essential for plant growth. It has been suggested that the energy saved by down-regulation of primary metabolism is diverted and used for defense responses [5, 6]. Nevertheless, up-regulation of primary metabolism also occurs during plant-pathogen interactions and is believed to be associated with signal transduction cascades that lead to plant defense responses [7].

Pathogen-triggered cellular responses involve massive transcriptional reprogramming within the host. Hormone signaling and transcription factors (TFs) are the two major facilitators of downstream defense responses in plants [8, 9]. Plant hormones as cellular signal molecules play key roles in regulating immune responses to invasion by microbial pathogens. Their signaling pathways are interconnected in a complex network providing plants with an enormous regulatory potential to rapidly respond to biotic stress while limiting the use of resources essential for basic metabolism [8]. According to their lifestyle, plant pathogens are generally divided into necrotrophs and biotrophs [10]. Necrotrophic pathogens first destroy host cells, often through the production of phytotoxins and cell-wall degrading enzymes, and then feed on dead tissues. Biotrophic pathogens feed on live tissues. Some plant pathogens, displaying both lifestyles depending on their life stage, are called hemi-biotrophs. *Fg* has been described as displaying a hemi-biotrophic life style in wheat [11]. Major plant hormones that regulate defense responses include salicylic acid (SA), jasmonic acid (JA) and ethylene (ET). Generally, SA plays a key role in defense against biotrophic pathogens, while JA and ET are critical to defense against necrotrophic pathogens [10]. The effective defense against biotrophic pathogens is largely due to programmed cell death in the host, and to the associated activation of defense responses regulated by SA–dependent pathways. Defense responses against necrotrophic pathogens are activated by JA and ET signaling.

Transcriptional reprogramming is governed by TFs and co-regulatory proteins organized in discrete transcriptional complexes [12]. Transcription factors are often sites of signal convergence and signal-regulated TFs act in concert with other context-specific TFs and transcriptional co-regulators to establish sensory transcription-regulatory networks required for plant immunity [9]. The TF families involved in plant immunity include AP2/ERF, bHLH, bZIP, MADS box, MYB, NAC, and WRKY [9, 13, 14]; their respective roles are reviewed in [9].

RNA sequencing (RNA-seq) technology has been very informative for transcriptomics studies. The recent release of the complete wheat genome sequence (pseudomolecules) and detailed annotations allowed exploratory analysis of DEGs associated with resistance and susceptibility against FHB, specifically in known FHB resistance QTL regions. We applied RNA-seq technology to study the transcriptomics response of four wheat genotypes (the FHB resistant Nyubai, Wuhan 1 and their progeny line HC374, and the FHB-susceptible Shaw) after inoculation with *Fg*.

## Results

**Fig. 1:**
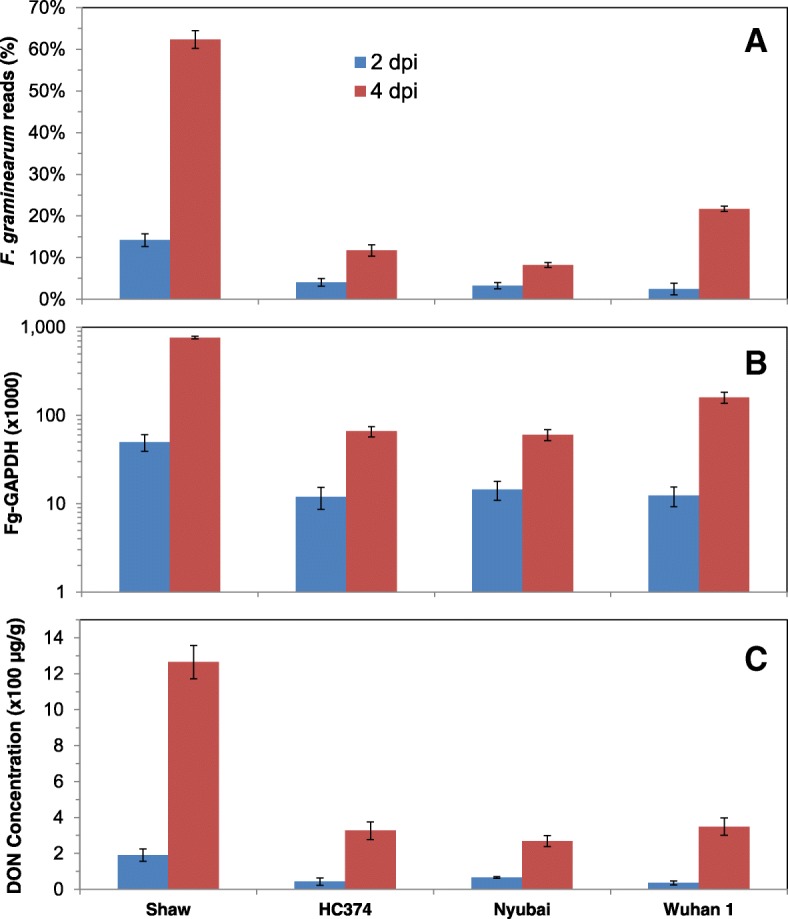
Level of *Fg* infection as estimated by proportion of *Fg* RNAs in RNA-seq reads (**a**), by accumulated level of *FG-GAPDH* RNA measured by RT-qPCR (**b**), and by DON concentration (**c**) across the samples. Error bar = one standard error of mean

RNA-seq data were acquired for Nyubai, Wuhan 1, HC374 and Shaw from water-treated (control) and *Fg*-inoculated spikelets at 2 and 4 days post inoculation (dpi). Paired-end reads only were considered in the mapping to the reference genome (average mapping rate of 96%). Results show that the proportion of pathogen transcripts in the wheat samples was highest in the susceptible genotype Shaw and lower in the three resistant genotypes, HC374, Nyubai and Wuhan 1, and increased from 2 to 4 dpi across all wheat genotypes; these results were confirmed by estimation of the fungal biomass, based on expression level of *Fg*-*GAPDH*, and by measurement of DON concentration (correlations of 0.99 and 0.98, respectively, Fig. 1).

**Fig. 2:**
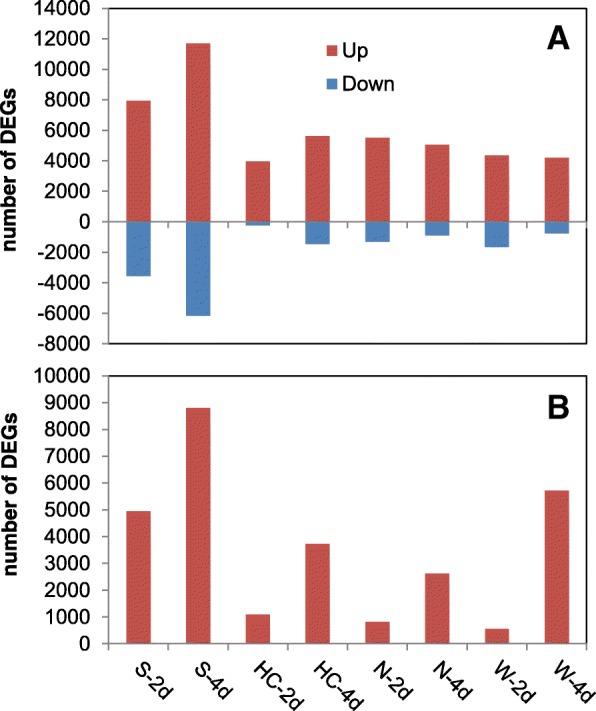
Total number of differentially expressed genes (DEGs) originating from wheat (**a**) and the pathogen (**b**), combining all DEG analyses. Up: upregulated, Down: down-regulated by *Fg*; 2d and 4d: 2 and 4 dpi; S: Shaw; HC: HC374; N: Nyubai; W: Wuhan 1

**Fig. 3:**
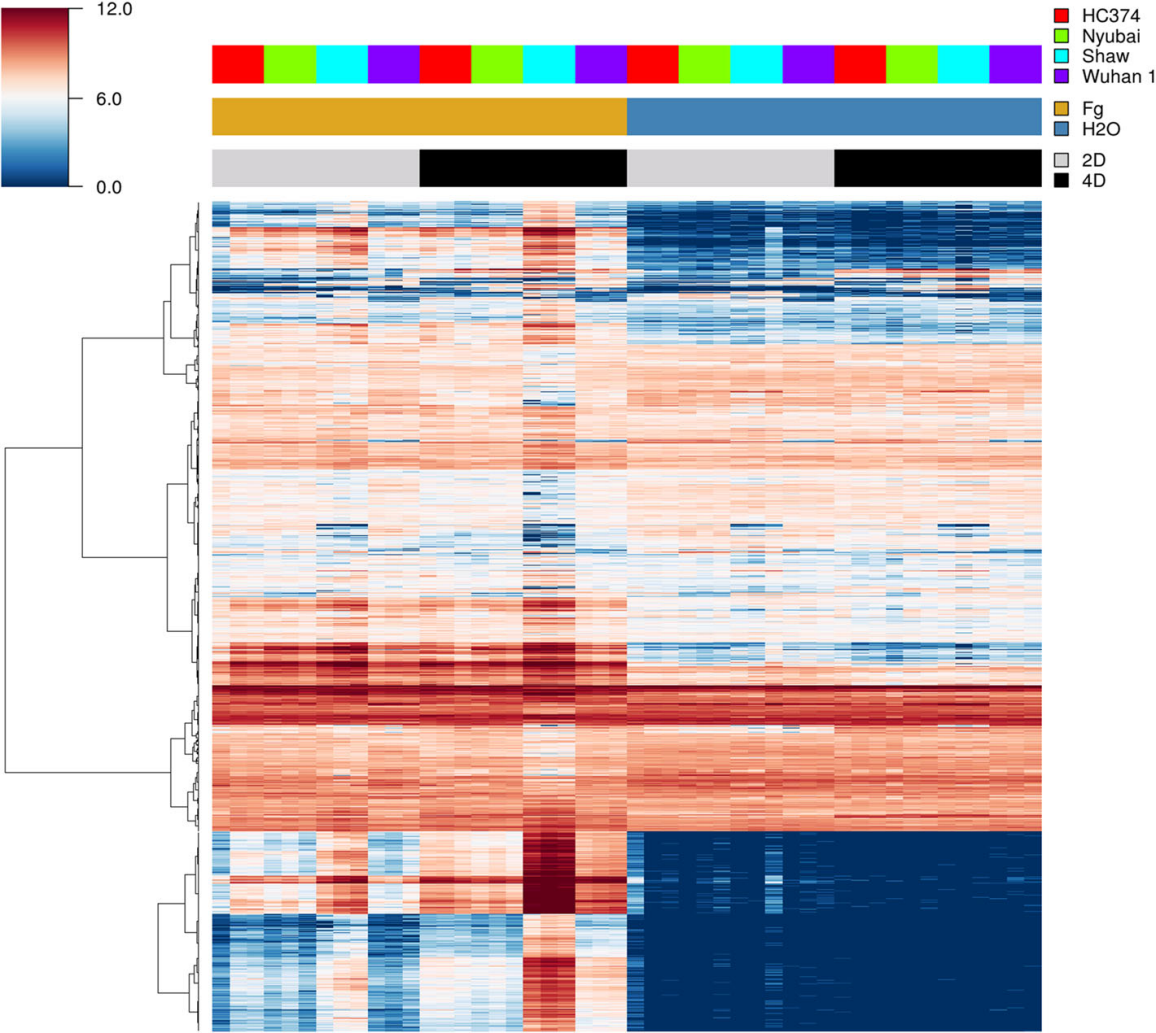
Global view of the DEG expression profiles between genotypes and treatments. The top dendrogram on the left represents 28,961 wheat genes and the bottom one 8811 *Fg* genes. Fg and H_2_O: treatments with *Fg* and water (control); 2d and 4d: 2 and 4 dpi

**Fig. 4:**
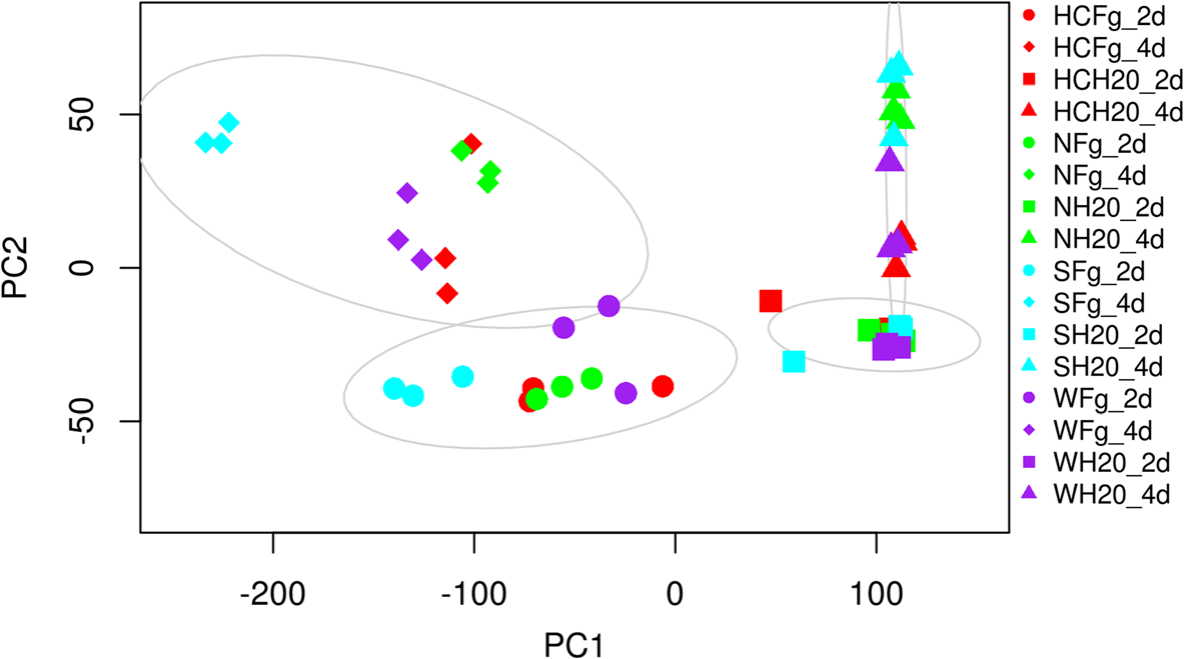
Principal component analysis of the wheat DEGs dataset based on the top 1000 most variable DEGs. PC1 explained 89% of variance and PC2 8%. The ellipses were 90% confidence intervals highlighting treatment/time clusters. Fg and H_2_O: treatments with *Fg* and water (control); 2d and 4d: 2 and 4 dpi; S: Shaw; HC: HC374; N: Nyubai; W: Wuhan 1

A total of 37,772 DEGs were identified: 28,961 from the wheat host and 8811 from the pathogen, with highest numbers in the susceptible host Shaw (Figs. 2, 3). Control samples were excluded for the differential analysis of *Fg* genes, because these samples theoretically didn’t contain any *Fg* mRNA. Clustering, correlation, differential expression feature extraction (DEFE) pattern and network analyses were done for wheat and pathogen genes separately. The principal component (PC) analysis of wheat DEGs (Fig. 4) revealed that differential expression was primarily driven by the *Fg* treatment (PC1), and secondly by duration of the treatment and genotype factors (PC2); these two PCs explained > 97% of variance.

For the purpose of this study, our analyses were focused on wheat genes. Using the recently developed differential expression feature extraction method (DEFE, Pan Y, Li Y, Liu Z, Surendra A, Wang L, Foroud NA, Goyal RK, Ouellet T, Fobert PR: Differential expression feature extraction and its application in wheat RNA-seq data analysis, forthcoming), four differential gene expression analyses were performed and seven DEFE pattern schemes were extracted (Table 1). For example, in comparisons between *Fg*-treated samples and the corresponding water-treated control samples, the pattern FW01020000 denotes up-regulation by *Fg* at 4 dpi in the susceptible Shaw, but down-regulation at 4 dpi in HC374, and no significant changes in Wuhan 1 or Nyubai. Each DEG was assigned a DEFE pattern ID from the seven pattern schemes (Additional file 1A, cols AV-BB).

**Table 1:** Differential expression feature extraction (DEFE) analyses

Set^a^	Modelled for	Treatment	DEFE series	Wheat DEGs	Pathogen DEGs
1	Effect of FHB	*Fg* vs. water	FW(S_2, S_4, H_2, H_4, N_2, N_4, W_2, W_4)	22,335	8809
2	FHB resistant vs. susceptible	*Fg*	FRS(H/S_2, H/S_4, N/S_2, N/S_4, W/S_2, W/S_4)	20,937	8803
		water	WRS(H/S_2, H/S_4, N/S_2, N/S_4, W/S_2, W/S_4)	7531	NA
3	Between resistant plants	*Fg*	FR(W/N_2, W/N_4, H/N_2, H/N_4, H/W_2, H/W_4)	10,637	4387
		water	WR(W/N_2, W/N_4, H/N_2, H/N_4, H/W_2, H/W_4)	5534	NA
4	Between two time points	*Fg*	FT(S_2/4, H_2/4, N_2/4, W_2/4)	6292	8331
		water	WT(S_2/4, H_2/4, N_2/4, W_2/4)	4287	NA

^a^Explanation of each set of comparisons

1. Comparison between each *Fg-*treated sample and the corresponding control sample. Where, the prefix “FW” stands for pairwise comparison between *Fg* and water treated (control) samples; for each comparison, S stands for Shaw, H for HC374, N for Nyubai and W for Wuhan 1

2. Comparisons of FHB resistant HC374, Nyubai, and Wuhan 1 with the susceptible Shaw in *Fg-*treated and control samples, respectively; where the second letter “R” in the prefix stands for FHB resistance genotypes

3. Pairwise comparison among the three FHB resistant genotypes in *Fg-*treated and control samples, respectively

4. Comparison between two time points of the same genotype in *Fg-*treated and control samples, respectively. Where, the second letter “T” stands for time

### Wheat DEGs highly correlated with *Fg* treatment

**Fig. 5:**
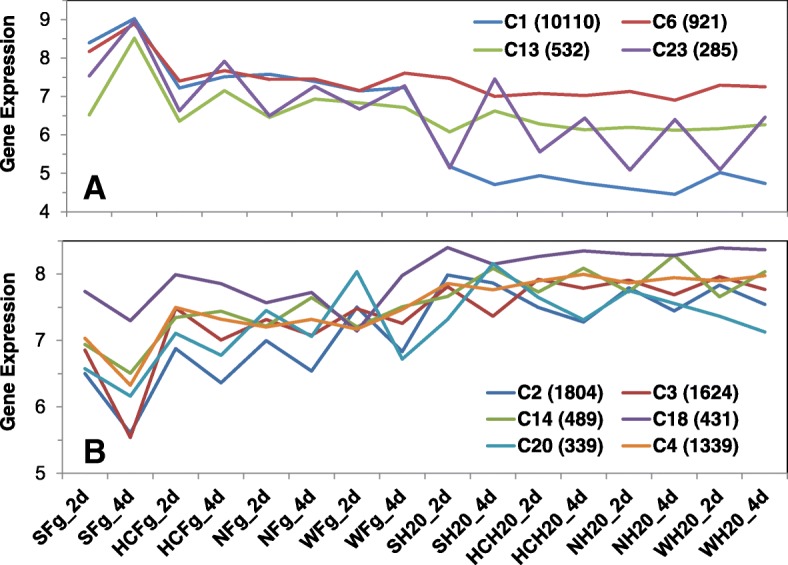
Average values of the expression profiles for the ten DEG clusters significantly up- (**a**) or down- (**b**) regulated by *Fg*. The numbers in the brackets are the numbers of genes in the respective clusters. Fg and H_2_O: treatments with *Fg* and water (control); 2d and 4d: 2 and 4 dpi; S: Shaw; HC: HC374; N: Nyubai; W: Wuhan 1

Forty clusters were identified among the 28,961 wheat DEGs (Additional file 1A, col AA). Pearson correlation analysis was performed between each cluster and FHB-related measurements including the percentage of RNA-seq reads from *Fg* (%Fg)*, Fg*-*GAPDH* and DON concentration (Fig. 1) and two experimental treatments (*Fg* inoculation and time after *Fg* inoculation). We define these FHB-related measurements and experimental treatments as phenotypic traits, collectively representing the effect of *Fg* infection. Four clusters, collectively containing 11,848 DEGs, were significantly up-regulated by *Fg* treatment as evidenced by the positive correlation (*p* ≤ 0.05) with all five phenotypic traits (*Fg* inoculation, time after *Fg* treatment, %*Fg* reads, *Fg*-*GAPDH* and DON levels), while another set of 6 clusters containing 6026 genes were down-regulated as evidenced by the negative correlation with these traits (Additional file 1D). The average expression profiles of the 10 clusters are illustrated in Fig. 5.

Similarly, a correlation analysis was performed between each individual gene and these phenotypic traits. For a gene to be qualified as significantly correlated (*p* ≤ 0.05) with a trait, it needed to be a member of the 10 clusters above, and the gene itself also had to be significantly correlated with the given trait. Each of the five FHB-related phenotypic traits covered nearly 60% of the DEGs; they collectively (union) accounted for 74% and jointly 41% of them (Table 2, Additional file 1A, cols BD, BE).

**Table 2:** Number of wheat DEGs significantly correlated with FHB related variables. Values for %Fg reads, Fg-GAPDH and DON are from Fig. 1

	FHB related variables						
	%*Fg* reads	*Fg*-*GAPDH*	DON	*Fg* treatment	Time after *Fg* treatment	Union	Joint
up	11,680	9932	11,563	10,761	11,182	12,963	7769
down	7345	6981	7352	5509	6086	8443	4148

**Fig. 6:**
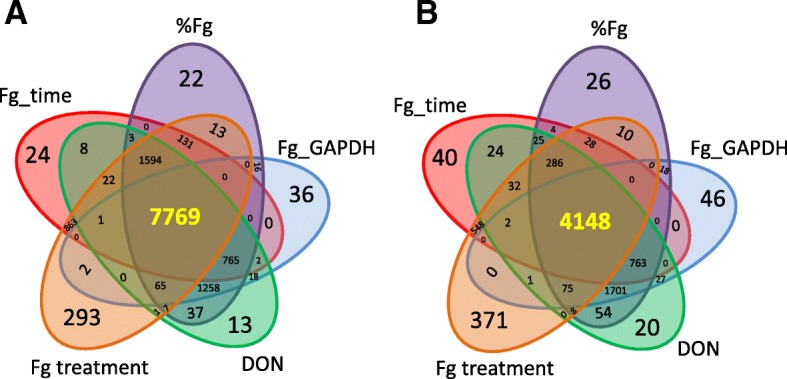
Numbers of DEGs correlated with the five FHB related traits, specific to each trait, common between 2, 3, 4, or all five traits. Values for %Fg, Fg_GAPDH and DON are from Fig. 1. **a** up-regulated DEGs; **b** down-regulated DEGs

**Fig. 7:**
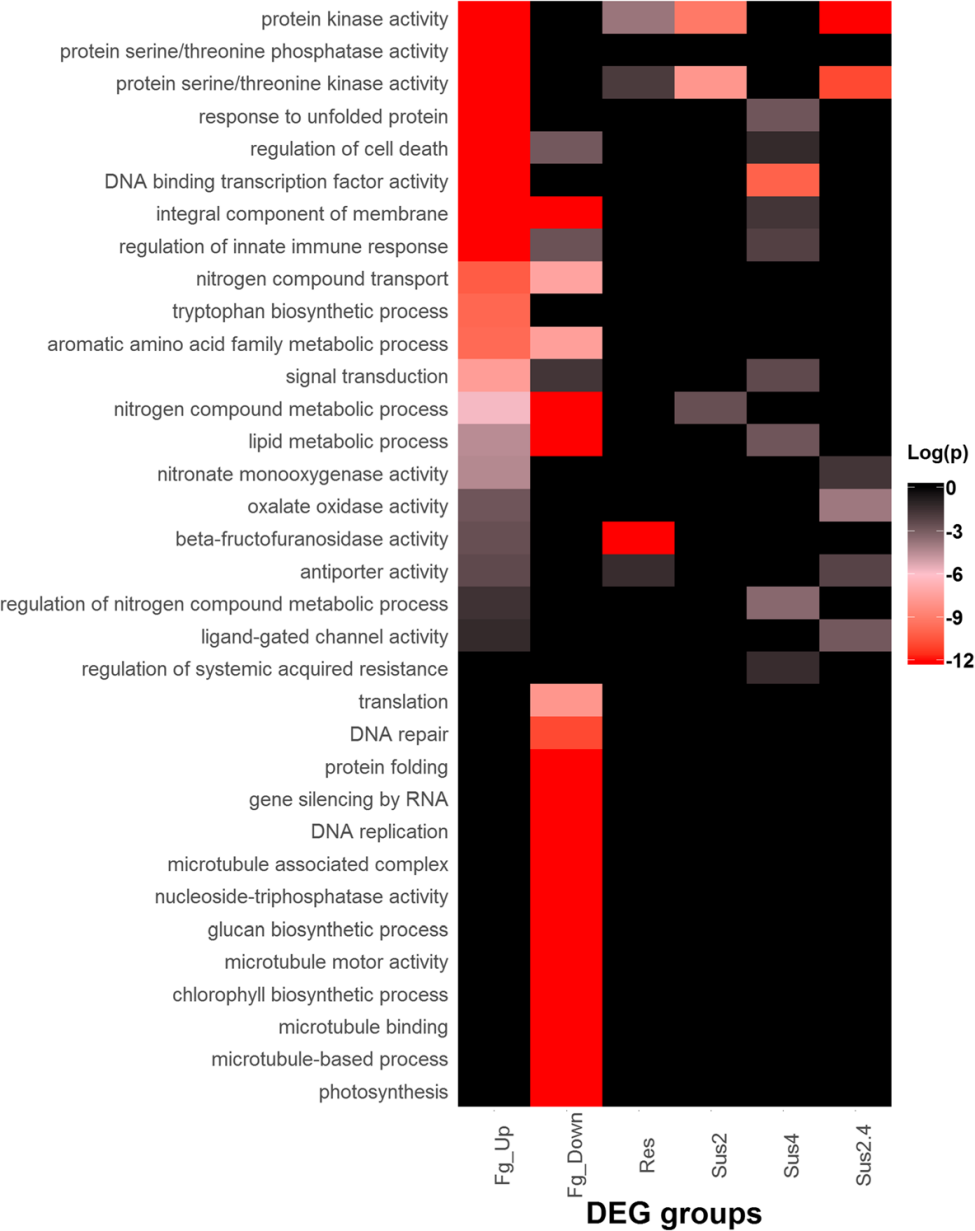
Gene ontology enrichment analysis of DEG groups up- or down-regulated by Fg (Fg_Up and Fg_Down) and genes associated with FHB resistance (Res) and susceptibility at 2 and/or 4 dpi (Sus2, Sus4, and Sus2.4)

**Fig. 8:**
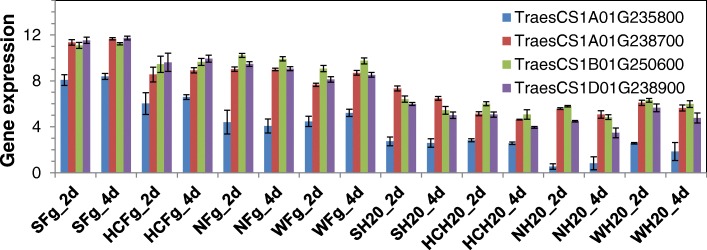
Gene expression profiles of nitronate monooxygenase genes. Fg and H_2_O: treatments with *Fg* and water (control); 2d and 4d: 2 and 4 dpi; S: Shaw; HC: HC374; N: Nyubai; W: Wuhan 1

Jointly considering the five phenotypic traits, there were 7769 up-regulated and 4148 down-regulated genes (Fig. 6). Gene ontology enrichment analysis indicates that the group of 7769 up-regulated genes was largely involved in gene regulation, including protein kinase activity and in particular serine/threonine kinases, serine/threonine phosphatase activity and transcription factor activity (Fig. 7). Key processes associated with biotic stress, such as regulation of cell death, response to unfolded protein and regulation of immune response, were also strongly affected. Changes associated with phytohormone pathways, including SA, JA and abscisic acid (ABA), were significant and will be discussed in more detail in a later section. Enrichment was also observed for the up-regulation of genes associated with aromatic amino acid metabolism, in particular tryptophan biosynthesis, and for genes associated with nitrogen compound transport and nitronate monooxygenase activity. Interestingly, only four genes (TraesCS1B01G250600, TraesCS1A01G235800, TraesCS1A01G238700, and TraesCS1D01G238900) in the wheat genome were annotated with nitronate monooxygenase activity; all of them were significantly associated with all five FHB-related traits (Fig. 8). More details are available in Additional file 2A.

The group of 4148 down-regulated genes was highly enriched with genes associated with microtubule-based processes including binding, motor activity and nucleoside-triphosphatase activity. It was also enriched in genes associated with primary metabolic processes, such as the nitrogen compound metabolic process, glucan biosynthetic process, chlorophyll biosynthetic process and photosynthesis (Fig. 7). DNA replication and repair, gene silencing by RNA, as well as translation and protein folding were also negatively impacted by *Fg* infection. More details are available in Additional file 2B.

The following sections examine the contribution of genes whose expression changed in response to *Fg* treatment, in order to gain better understanding of the molecular response of wheat to *Fg* infection.

### DEGs potentially associated with FHB resistance

**Fig. 9:**
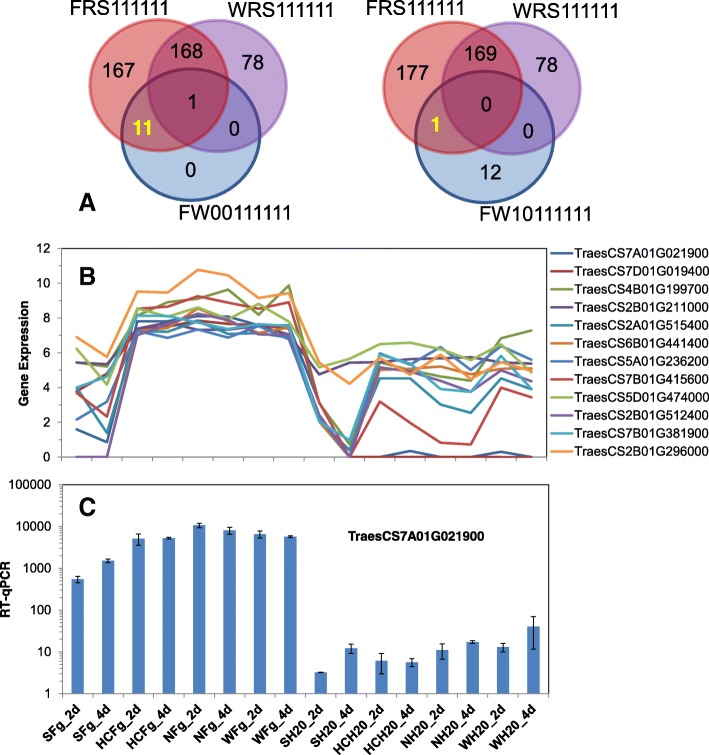
Genes putatively associated with FHB resistance. **a** 12 genes upregulated by *Fg* treatment across the resistant Nyubai, Wuhan 1 and HC374, but not in the susceptible Shaw were identified by the joint DEFE patterns of FW00111111∩ (FRS111111¬WRS111111) or FW10111111∩(FRS111111¬WRS111111). Refer to Table 1 for the meaning of the DEFE patterns. **b** expression profiles of the 12 genes putatively associated with FHB resistance. **c** expression profile by RT-qPCR for GST (TraesCS7A01G021900). Fg and H_2_O: treatments with *Fg* and water (control); 2d and 4d: 2 and 4 dpi; S: Shaw; HC: HC374; N: Nyubai; W: Wuhan 1

There were a very small number of genes (12) with a DEFE feature pattern FW00111111, which identifies genes up-regulated by *Fg* across all three resistant genotypes at both time points, but with no significant change in the susceptible Shaw. All genes in this group have the same DEFE pattern FRS111111 showing significantly higher expression in the resistant genotypes as compared with the susceptible Shaw. One gene having a feature pattern WRS111111, indicating similar transcriptional profile in the control sample, was removed from this group. The combined DEFE feature pattern FW00111111∩(FRS111111¬WRS111111) includes 11 genes consisting of three glutathione S-transferase (*GST*), five protein kinases, a purple acid phosphatase and two membrane proteins (Table 3, Fig. 9). Additionally, another gene (TraesCS2B01G296000, a *MatE* transmembrane transporter) was included in the combined DEFE feature pattern FW10111111∩(FRS111111¬WRS111111). Although it was upregulated in Shaw at 2 dpi, the extent of change (log2FC = 1.5) was minor as compared to the three resistant genotypes (log2FC > 3.5); the differences in gene expression between a resistance genotype and the susceptible Shaw (FRS111111) were at log2FC > 2 (Table 3). The two *GST* from chromosome 7 are possible homolog genes. The expression levels of the 12 genes were mostly correlated with pathogen inoculation in the three resistant genotypes. The expression of these genes in the susceptible Shaw was much lower, even after challenge by the pathogen, showing a highly significant negative correlation (*p* < 0.002) (Fig. 9, Additional file 3A).

**Table 3:** Genes upregulated by *Fg* treatment across the resistant HC374, Nyubai, and Wuhan 1, but not in the susceptible Shaw

Gene ID	Human-Readable-Description	log2 Fold Changes							
		S_2	S_4	H_2	H_4	N_2	N_4	W_2	W_4
TraesCS7A01G021900	Glutathione S-transferase	NS^a^	NS	9.6	8.7	10.4	9.7	8.6	9.4
TraesCS7D01G019400	Glutathione S-transferase	NS	NS	9.3	9.4	10.3	9.5	9.6	9.4
TraesCS4B01G199700	Glutathione-S-transferase	NS	NS	2.7	3.9	4.5	5.3	1.4	2.6
TraesCS2A01G515400	Leucine-rich repeat receptor-like protein kinase	NS	NS	2.7	2.7	4.9	4.7	2.6	3.0
TraesCS6B01G441400	Leucine-rich repeat receptor-like protein kinase	NS	NS	2.3	2.4	3.4	3.1	2.1	2.4
TraesCS7B01G415600	Protein tyrosine kinase with lectin domain	NS	NS	5.3	6.8	9.1	8.9	4.5	5.4
TraesCS2B01G512400	Serine/threonine-protein kinase with S-locus glycoprotein domain	NS	NS	1.9	2.7	3.9	4.2	2.1	2.7
TraesCS2B01G211000	plasma membrane protein hyccin, a regulator of phosphatidylinositol phosphorylation	NS	NS	1.9	2.1	1.6	1.6	2.1	1.7
TraesCS5A01G236200	Phosphatidylinositol-4-phosphate 5-kinase	NS	NS	1.3	1.5	1.0	1.9	1.2	1.2
TraesCS5D01G474000	Purple acid phosphatase	NS	NS	2.1	1.5	2.4	2.4	2.3	2.9
TraesCS2B01G296000	MatE transmembrane transporter	1.5	NS	3.8	4.7	4.9	6.0	3.7	4.5
TraesCS7B01G381900	transmembrane protein of unknown function	NS	NS	2.2	2.7	3.8	3.7	1.8	3.7

^a^NS = not significant

**Fig. 10:**
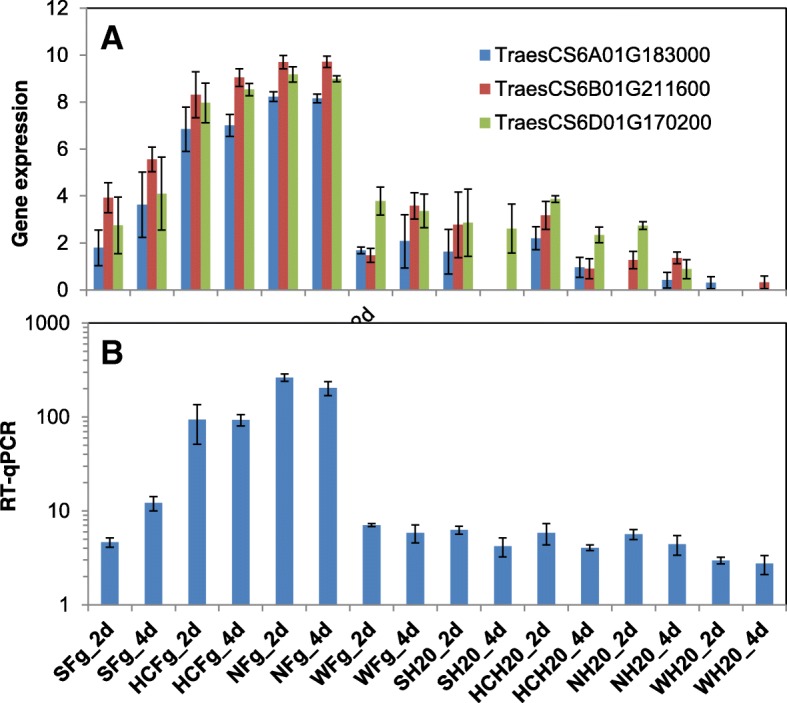
Expression profiles of sesquiterpene synthases. **a** RNA-seq data of the three homologs. **b** cumulative expression profile by RT-qPCR for the three sesquiterpene synthases (gene specific assays could not be designed due to high sequence similarity among the three genes). Fg and H_2_O: treatments with *Fg* and water (control); 2d and 4d: 2 and 4 dpi; S: Shaw; HC: HC374; N: Nyubai; W: Wuhan 1

The small number of commonly up-regulated genes among the resistant genotypes can be explained at least in part by the genetic relationship between wheat genotypes. Nyubai and Wuhan 1 are genetically different from each other while HC374 was derived from a cross between the two. There were more genes commonly associated with FHB resistance between Nyubai and HC374 (17) than between Wuhan 1 and HC374 (5). To study the similarity between Nyubai and HC374 and their difference with Wuhan 1, three DEFE patterns: FW00010100, FW00111100, FW00101000 and their combination with Sets 2 and 3 comparisons were investigated (Tables 4, 5, 6; Additional file 3B). For example, three sesquiterpene synthase homolog genes were found to have similar expression between Nyubai and HC374, but not Wuhan 1 (Fig. 10).

**Table 4:** DEGs upregulated by *Fg* treatment in HC374 and Nyubai, but not in Wuhan 1, neither in Shaw

Gene_ID	Human-Readable-Description	log2 Fold Changes							
		S_2	S_4	H_2	H_4	N_2	N_4	W_2	W_4
at 2 dpi alone: FW00101000∩(FRS101000¬WRS101000)									
TraesCSU01G181100	Ice recrystallization inhibition protein-like protein	NS^a^	NS	3.6	NS	7.3	NS	NS	NS
TraesCS1B01G044200	NBS-LRR-like resistance protein	NS	NS	1.0	NS	1.1	NS	NS	NS
TraesCS3B01G339700	Peroxidase	NS	NS	9.1	NS	10.3	NS	NS	NS
TraesCS3B01G421700	Transcription initiation factor TFIID subunit 9	NS	NS	1.3	NS	1.1	NS	NS	NS
at both 2 and 4 dpi: FW00111100∩(FRS111100¬WRS111100)									
TraesCS3B01G261500	Elongation factor	NS	NS	1.0	1.3	1.0	2.1	NS	NS
TraesCS6B01G442600	Leucine-rich repeat receptor-like protein kinase family protein	NS	NS	6.2	6.1	7.5	6.0	NS	NS
TraesCS6D01G050800	Leucine-rich repeat receptor-like protein kinase family protein	NS	NS	1.5	1.8	2.0	2.2	NS	NS
TraesCS3B01G485100	MatE transmembrane transporter	NS	NS	1.4	1.9	3.7	4.4	NS	NS
TraesCS6B01G444300	Mitochondrial metalloendopeptidase OMA1	NS	NS	2.8	4.9	5.7	6.2	NS	NS
TraesCS1B01G048100	Phenylalanine ammonia-lyase	NS	NS	5.5	4.1	6.7	5.5	NS	NS
TraesCS5B01G267500	plant/protein (Protein of unknown function, DUF538)	NS	NS	5.7	7.6	10.5	10.5	NS	NS
TraesCS7B01G133700	Protein O-linked-mannose beta-1,4-N-acetylglucosaminyltransferase 2	NS	NS	2.0	2.5	2.6	2.6	NS	NS
TraesCS6A01G417900	Receptor protein kinase, putative	NS	NS	7.1	7.7	7.9	7.4	NS	NS
TraesCS1B01G022000	Serine/threonine-protein kinase	NS	NS	2.1	2.3	3.2	3.7	NS	NS
TraesCS6A01G183000	Sesquiterpene synthase	NS	NS	4.8	6.6	10.5	8.9	NS	NS
TraesCS6B01G211600	Sesquiterpene synthase	NS	NS	5.1	8.6	8.9	8.7	NS	NS
TraesCS6D01G170200	Sesquiterpene synthase	NS	NS	4.0	6.2	6.4	8.6	NS	NS

^a^NS = not significant

**Table 5:** DEGs upregulated by *Fg* treatment in HC374 and Wuhan 1, but not in Nyubai, neither in Shaw

Gene_ID	Human-Readable-Description	log2 Fold Changes							
		S_2	S_4	H_2	H_4	N_2	N_4	W_2	W_4
at both 2 and 4 dpi: FW00110011∩(FRS110011¬WRS110011)									
TraesCS2B01G554800	Reticulocyte-binding protein 2 homolog a	NS^a^	NS	2.7	3.3	NS	NS	1.3	1.9
at 4 dpi alone: FW00010001∩(FRS010001¬WRS010001)									
TraesCS5B01G523100	Cysteine protease, putative	NS	NS	NS	8.3	NS	NS	NS	9.3
TraesCSU01G128400	Germin-like protein	NS	NS	NS	8.8	NS	NS	NS	9.4
TraesCS7D01G542800	Phosphatidylinositol-4-phosphate 5-kinase family protein	NS	NS	NS	2.5	NS	NS	NS	2.0
TraesCS2A01G536300	Serine/threonine-protein kinase	NS	NS	NS	2.4	NS	NS	NS	1.4

^a^NS = not significant

**Table 6:** DEGs upregulated by *Fg* treatment in the two parents Nyubai and Wuhan 1, but not in their progeny HC374, neither in Shaw

Gene_ID	Human-Readable-Description	log2 Fold Changes							
		S_2	S_4	H_2	H_4	N_2	N_4	W_2	W_4
at both 2 and 4 dpi: FW00001111∩(FRS001111-WRS001111)									
TraesCS2B01G572300	Blue copper protein	NS^a^	NS	NS	NS	8.4	9.6	7.4	11.3
TraesCSU01G212400	Blue copper protein	NS	NS	NS	NS	10.3	9.1	9.4	10.9
TraesCS2B01G519000	Cytochrome P450 family protein	NS	NS	NS	NS	9.4	8.8	4.2	6.5
TraesCS2B01G083700	Glycosyltransferase	NS	NS	NS	NS	2.3	2.3	1.8	1.3
TraesCSU01G205900	O-methyltransferase-like protein	NS	NS	NS	NS	9.6	10.2	9.4	9.4
TraesCSU01G246000	O-methyltransferase-like protein	NS	NS	NS	NS	10.4	9.7	9.1	9.0
TraesCSU01G074400	plant/protein (Protein of unknown function, DUF538)	NS	NS	NS	NS	9.0	6.7	6.6	7.7
at 4 dpi: FW00000101∩(FRS000101¬WRS000101)									
TraesCS5D01G557900	carboxyl-terminal peptidase, putative (DUF239)	NS	NS	NS	NS	NS	5.0	NS	2.2

^a^NS = not significant

Other genes associated with FHB resistance but with distinct expression profiles in different genotypes are listed in Additional file 3C. They illustrate some of the differences in the overall defense response to *Fg* infection in each genotype. For example, there were roughly similar proportions of genes annotated as involved in the regulation of gene expression, defense, secondary metabolism, protein degradation and transport among the 107 DEGs unique to Wuhan 1, while about half of the 50 DEGs unique to Nyubai were involved in gene regulation, a quarter in secondary metabolism and very few in the other 3 functional categories. Among the 107 DEGs unique to Wuhan 1, 92 were up-regulated only at 2 dpi and the other 15 only at 4 dpi, but none were up-regulated at both time points. Among the 50 Nuybai DEGs, 46 were up-regulated at 2 dpi and 19 were also up-regulated at 4 dpi. Thus, DEGs in Wuhan 1 were transiently up-regulated by *Fg*, while many in Nyubai were much more strongly up-regulated by the pathogen at both time points. Only 21 DEGs had a distinct expression profile for HC374, with most (18/21) showing higher up-regulation by *Fg* at 4 dpi only. Interestingly, nine of these genes are annotated as acid beta-fructofuranosidases (Fig. 7; Additional file 2C, Additional file 3C). These genes are part of a small protein family containing distinct members co-localized on chromosomes 6A, B and D.

RT-qPCR analysis was performed for a subset of the genes associated with resistance mentioned above. Similar expression profiles were obtained when the genes tested had no close homolog (*GST*, Traes7A01G021900, Fig. 9c) or when all of the homologs had a similar expression pattern (sesquiterpene synthase TraesCS6A01G183000 and homologs, Fig. 10).

### DEGs potentially associated with FHB susceptibility

**Fig. 11:**
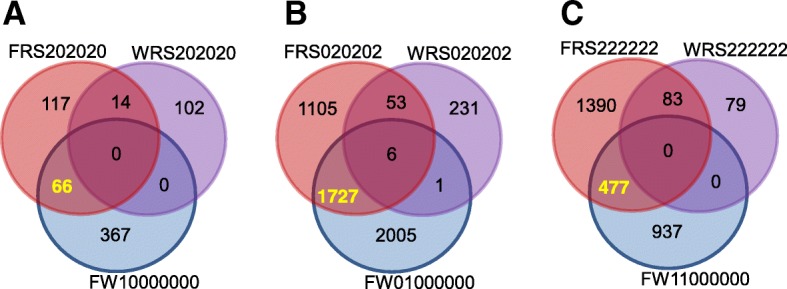
DEGs putatively associated with FHB susceptibility. Frequency distribution at 2 dpi (**a**), 4 dpi (**b**), and both time points (**c**). The highlighted numbers in the Venn diagrams are considered in the text. Refer to Table 1 for the meaning of the DEFE patterns

In comparisons between *Fg*-treated samples and their controls, the number of DEGs of both host and pathogen were highest in the susceptible host Shaw, consistent with the *Fg* biomass (estimated by *Fg*-*GAPDH* expression), mRNA abundance, and DON concentration (Figs. 1, 2). In wheat, there were 3739 genes uniquely up-regulated at 4 dpi in Shaw (FW01000000 = 3739), which was the most frequent DEFE feature pattern of the series (Additional file 1B). In *Fg-*treated samples, there were 2891 genes expressed unanimously lower at 4 dpi in all three resistant genotypes than in the susceptible Shaw (FRS020202 = 2891). This pattern had the highest frequency in that series as well. These two interesting feature patterns prompted us to investigate DEGs potentially associated with FHB susceptibility in Shaw. FRS020202 confirms FW01000000 in up-regulation by *Fg* treatment at 4 dpi only in Shaw. There were a smaller number of genes (291) with a DEFE feature pattern of WRS020202, which represents innate difference between all three FHB resistant genotypes and the susceptible Shaw without *Fg* treatment. Combining these three feature patterns in the formula FW01000000∩FRS020202¬WRS020202 revealed a group of 1727 genes putatively associated with susceptibility in Shaw at 4 dpi (Fig. 11). Similarly, there were 66 genes with a formula of FW10000000∩FRS202020¬WRS202020 (same as previous formula, but at 2 dpi) putatively associated with susceptibility at 2 dpi in Shaw and 477 genes of FW11000000∩FRS222222¬WRS222222 putatively associated with susceptibility in Shaw at both 2 and 4 dpi.

Gene Ontology enrichment analysis indicated that the 66 genes uniquely up-regulated in the susceptible genotype at 2 dpi by *Fg* were highly enriched with genes encoding for protein kinase activity, particularly protein serine/threonine kinases (Fig. 7). Changes in nitrogen compound metabolic process, the other most important enriched category, were relatively modest (Additional file 2D).

**Fig. 12:**
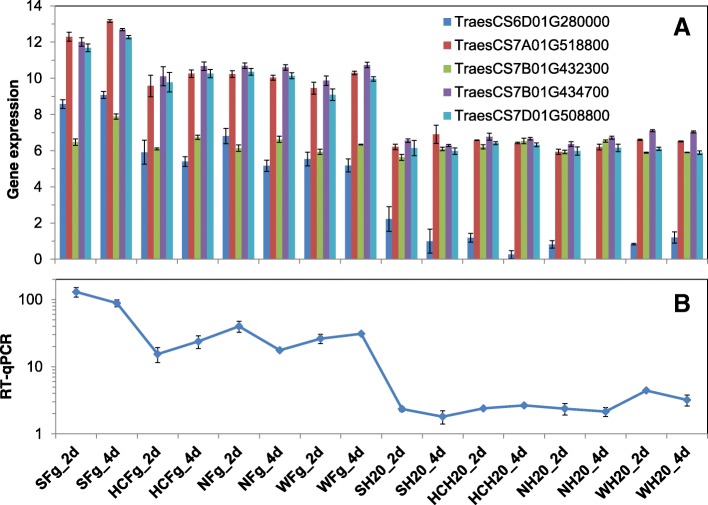
Expression profiles of the five *NFXL1* genes. **a** in the RNA-seq dataset; **b** cumulative expression of the four genes on chromosome 7 by RT-qPCR (gene specific assays could not be designed due to high sequence similarity among the four genes). Fg and H_2_O: treatments with *Fg* and water (control); 2d and 4d: 2 and 4 dpi; S: Shaw; HC: HC374; N: Nyubai; W: Wuhan 1

The group of 1727 genes up-regulated only in Shaw at 4 dpi, was highly enriched with genes involved in regulation of gene expression, including ET-responsive, *WRKY*, *Myb*, *bZIP*, *NAC*-domain containing and other types of transcription factors (Additional file 2E). This group also includes the NF-X1-type zinc finger protein *NFXL1*, which has previously been shown to contribute to FHB susceptibility in wheat [15] (Fig. 12). These changes correlated with the regulation of several processes, including nitrogen compound and lipid metabolism, signal transduction and composition of membranes (Fig. 7).

The group of 477 genes associated with susceptibility of Shaw at both 2 and 4 dpi was enriched in genes coding for protein kinase activity (Fig. 7), as observed at 2 dpi; however the up-regulation in Shaw of this larger number of kinase genes lasted through the two time points sampled. This group was also enriched in genes associated with oxalate oxidase activity and transport, especially *MatE* antiporters. More details are provided in Additional file 2F.

To further understand the dynamics of changes in regulation of gene expression in Shaw, the distribution of genes in key functional categories associated with expression regulation was examined using their gene description (Additional file 1A, cols C, BG-BI). This allowed the inclusion of genes without GO annotations. A shift can be observed in the types of kinases up-regulated uniquely in Shaw during the experiment (Table 7). A majority of the leucine rich repeat receptor kinases (LRR-RKs) were up-regulated in Shaw at 2 dpi, while most other types of kinases were up-regulated either at 4 dpi or at both time points. It is of note that only two mitogen-activated protein (MAP) kinases were up-regulated uniquely in Shaw among the 53 MAP kinases differentially expressed after *Fg* treatment. The majority of the transcription factors were uniquely up-regulated in Shaw at 4 dpi. Another group of genes involved in regulation via the proteasome, the F-box containing proteins, were also up-regulated in Shaw at 4 dpi or at both time points.

**Table 7:** Dynamic of expression for selected regulatory genes up-regulated only in the susceptible genotype Shaw

Predicted Function	Type	2 dpi	4 dpi	Both time points
Kinase	Receptor	1	27	29
	Leucine-rich repeat receptor	14	2	8
	*MAP*	0	2	0
	Serine/threonine	6	19	20
	Others	3	60	27
	Metabolism	0	23	2
Transcription factor	*WRKY*	0	21	4
	*Myb*	2	24	8
	Ethylene-responsive	1	20	3
	Others	0	49	11
F-box protein		1	58	86

### Gene association network analysis

**Fig. 13:**
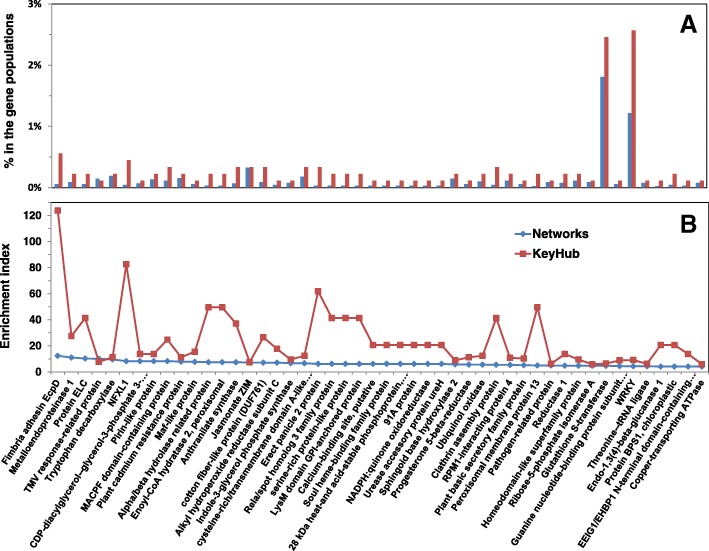
Enrichment of gene groups in the association networks and in key hub gene populations. **a** proportion in the gene populations, **b** enrichment index. Details are in Additional file 4. The color legend in panel b is for both panels

The top 1% of the similarity matrix (Eq. 1 in Methods) was considered in a network consisting of 8946 wheat DEGs connected with 4,328,705 edges. The majority of network nodes were from the cluster C1, which exclusively occupied the top 53% of nodes (4766 genes). This cluster included a large number of genes that were strongly up-regulated by *Fg* infection, with the up-regulation in Shaw being the largest. The top 10% of nodes (895 genes, highlighted in Additional file 1A, col AB) in the networks were considered key hub genes. The lowest number of immediate neighbors (directly connected genes) among the key hub genes was 3030. The top node (TraesCS1D01G429900), having 6553 immediate neighbors, was a *WRKY* transcription factor (TF). There were 23 *WRKY* TF genes among the top 10% key hub genes and 109 in the entire network. An enrichment index (defined in the method section) was applied to illustrate the extent of enrichment of interesting groups of genes. For example in the case of *WRKY* TFs, there were 23 (2.57% = 23/895) in the key hub gene population, 109 (1.22% = 109/8946) in the entire network, 145 (0.50% = 145/28961) in the DEGs and 311 (0.28% = 311/110790) in the entire wheat genome; thus the enrichment index (*E*) for *WRKY* TFs was 1.78, 4.34, 9.15 in the DEGs, network and key hub gene populations, respectively. The *WRKY* TFs group was one of the two groups with the highest percentage of enrichment (Figs. 13a, Additional file 4). Glutathione S-transferases (*GST*) were also highly enriched, with 22 (*E* = 6.35) as key hub genes and 162 (*E* = 4.68) in the entire network (Fig. 13). Among the 8946 DEGs in the networks, 5340 were part of the *Fg* up-regulated group of genes (Fig. 6a, Additional file 4B) and 1019 were putatively associated with FHB susceptibility (Fig. 11).

The groups of genes annotated as fimbria adhesin *EcpD*, *NFXL1* and erect panicle 2 protein were among highly enriched groups (Fig. 13b); fimbria adhesin *EcpD* and *NFXL1* were up-regulated by *Fg* infection. There are only six *NFXL1* genes in the entire wheat genome, of which five were differentially expressed, and four were identified as key hub genes (Figs. 12, 13b). The six genes annotated as erect panicle 2 protein formed two subgroups with different expression profiles and orthologs (Additional file 5).

Collectively, among the 2270 DEGs putatively associated with FHB susceptibility, over 66% were in the group of *Fg* up-regulated genes (Fig. 6a) and the majority (60%) was in the C1 cluster (Additional file 1A, cols AA, BG-BI). Among genes associated with FHB susceptibility, many (175) were also key hub genes, including 12 F-box proteins, 2 *WRKY* TFs, 2 JA *ZIM*, and 2 glutathione S-transferases (Additional file 4B).

### DEGs encoding pathogenesis-related proteins

Of the 235 genes in the wheat genome known to be closely related to plant pathogenesis, based on the wheat IWGSC RefSeq v1.0 gene function annotation, description, gene ontology fields, and manual curation, 57 were DEGs in the analyzed dataset (Additional file 6). These included 29 pathogenesis-related protein 1 (*PR1*), 2 *PR1–1*, 9 *PR4*, 7 thaumatin-like, and other genes detailed in Additional file 6. *PR1* genes are known to be associated with the SA pathway while *PR4* and thaumatin genes are associated with JA and/or ET pathway. In the list of DEGs, 13 were putatively associated with FHB susceptibility and none with FHB resistance (Additional file 6, cols BD & BE). There were 33 PR genes that were also nodes in the gene association network. Two *PR1* (TraesCS5B01G442900, TraesCS5D01G446800) were considered key hub genes and were associated with *Fg* challenge, but not with FHB susceptibility or resistance. All *PR1*, *PR1–1* and *PR-4* genes were up-regulated by *Fg*, but none of them were expressed higher in any resistant genotype than in the susceptible Shaw.

### Genes related with hormonal effects in response to *Fg* challenge

Through an ortholog search in *Arabidopsis thaliana*, *Brachypodium distachyon*, *Oryza sativa* Japonica and *Zea mays*, and using the current and previous versions of wheat genome annotations (Survey genome version 2.2 and TGACv1 in Ensembl Plants), 542 wheat DEGs were identified as involved in phytohormone pathways (Additional file 7). Some annotations were assigned using orthologs, to be more specific. Nearly half of these hormone pathway genes (245) were involved in the network described above and 24 were key hub genes. There were significantly more DEGs involved in auxin, ET, and JA pathways compared with ABA, SA, cytokinin, and gibberellin pathways (Table 8). The majority of the DEGs associated with the JA and ET response pathways were up-regulated by *Fg*, with a higher level of expression in the susceptible Shaw (Table 9). The only hormone biosynthetic pathway with multiple DEGs was the JA pathway. Of the 357 DEGs up-regulated by *Fg* infection, 80 were associated with FHB susceptibility (Additional file 7, cols BO-BQ); only two were associated with FHB resistance: auxin transport protein TraesCS6B01G198200 in HC374 and ET-responsive transcription factor TraesCS4D01G298600 in Wuhan 1.

**Table 8:** Number of DEGs involved in phytohormone pathways

Hormone	SA	JA	ET	Auxin	Cytokinin	Gibberellin	ABA
Freq	44	98	180	170	30	27	69

**Table 9:** Significant DEGs involved in the phytohormone pathways up or down regulated by *Fg* treatment

	Up	Down
Auxin	Auxin efflux carrier family proteins	Auxin influx transporter
	Auxin-induced in root cultures protein 12	Auxin efflux carrier components
	Auxin-responsive protein	Auxin response factor
	GH3.3	Auxin-responsive protein
	Indole-3-glycerol phosphate synthase	Early auxin response protein
	SAUR-like auxin-responsive family protein	
Ethylene	1-aminocyclopropane-1-carboxylate synthase, ACS6	Divalent metal cation transporter MntH 2
	Ethylene insensitive 3	Ethylene insensitive 2 transporter
	Ethylene insensitive 3-like protein	
	Ethylene-responsive transcription factor	
	Mitochondrial carrier family	
	Multiprotein-bridging factor, MBF1C	
JA	12-oxophytodienoate reductase	4-coumarate-CoA ligase family protein
	12-oxophytodienoate reductase-like protein	
	Accelerated cell death 11	
	Allene oxide cyclase	
	Allene oxide synthase	
	Jasmonate *ZIM* domain proteins	
	Lipoxygenases	
	Molybdopterin biosynthesis protein CNX1	
ABA	ABA-responsive binding factor	ABA deficient 2, ABA2
	Abscisic acid receptor	ABA deficient 1, ABA1
	cytochrome P450 family ABA 8′-hydroxylase	
	GRAM domain-containing protein / ABA-responsive	
	Molybdenum cofactor sulfurase (ABA3)	
	RING/U-box superfamily protein	
SA	Accelerated cell death 11, ACD11	ATP synthase delta-subunit
	LPS-induced tumor necrosis factor alpha factor	Guanine nucleotide-binding protein subunit alpha-like protein
	NPR1	
	NRR repressor homolog 1	
	Phytoalexin-deficient 4–1 protein (PAD4)	
	Salicylate o-methyltransferase	
	Stress-associated protein 12, SAP12	
	Mitogen-activated protein kinase, MPK3	
Cytokinin	Anamorsin homolog	L-galactono-1,4-lactone dehydrogenase
	Cytokinin oxidase/dehydrogenase	
	Cytokinin riboside 5′-monophosphate phosphoribohydrolase	
Gibberellin	Gibberellic acid methyltransferase 2	Gibberellin regulated proteins
	Gibberellin oxidases	
	Gibberellin receptor GID1a	

In the auxin signaling pathway, DEGs associated with regulation of IAA homeostasis included up-regulated efflux carrier family proteins and indole-3-acetic acid-amido synthetase GH3.3, and down-regulated influx transporters and efflux carrier components (Table 9). Those DEGs were respectively up- or down-regulated by *Fg* in all four genotypes, with a stronger differential expression in Shaw than in the resistant genotypes. The 17 *SAUR*-like auxin-responsive family proteins, all up-regulated by *Fg*, were heavily involved in the network with an average connectivity of 1738. Nine of the *SAUR*-like DEGs were associated with FHB susceptibility. In addition, 25 of the 41 auxin response factors and 13 of the 35 auxin-responsive proteins were down-regulated after *Fg* infection.

In the ET signaling pathway, DEGs annotated as positive regulators of the ET response pathway, ethylene-insensitive 3 and multiprotein-bridging factor MBF1 were up-regulated by *Fg* and expressed higher in the susceptible Shaw than any of the three resistant genotypes (Table 9, Additional file 7). *MBF1C* DEGs were also well connected in the network. One DEG (TraesCS2A01G396400, 1-aminocyclopropane-1-carboxylate synthase) was associated with ET biosynthesis; it was significantly up-regulated in all genotypes by *Fg* infection, and expressed at a higher level in Shaw.

Enzymes associated with JA biosynthesis, including allene oxide cyclase and synthase and 12-oxophytodienoate reductase, were up-regulated by *Fg*, with higher expression in FHB susceptible Shaw (Table 9, Additional file 7). Seven lipoxygenases were generally up-regulated by *Fg* in one or more genotypes. Three DEGs encoding LOX3/4 (TraesCS4A01G009400, TraesCS4D01G294100, and TraesCS2B01G333600) were highly involved in the association network. In the JA signaling pathway, the 38 DEGs encoding various JA *ZIM* domain proteins were all up-regulated by *Fg* and generally expressed at higher levels in Shaw than in the three FHB resistant genotypes. Five were associated with FHB susceptibility.

Many of the DEGs associated with ABA were up-regulated by *Fg* and expressed higher in Shaw. These included genes associated with regulation of ABA biosynthesis (ABA3), homeostasis (ABA 8′-hydrolase) and signaling (RING/U-box protein) (Table 9, Additional file 7). Of the five genes encoding ABA-responsive binding factor that were uniformly up-regulated by *Fg* and expressed progressively higher at 4 dpi in Shaw, four were also associated with FHB susceptibility.

Most of the DEGs associated with the SA pathway were up-regulated by *Fg* infection and showed a stronger response in Shaw (Table 9, Additional file 7). This included *NPR1*, salicylate o-methyltransferase, two *NRR* repressor homolog 1 genes, a receptor-like protein kinase, a putative protein kinase and accelerated cell death 11 (*ACD11*), which were all associated with FHB susceptibility. Three phytoalexin-deficient (PAD) 4–1 protein genes orthologous to *Arabidopsis PAD4* (AT3G52430), and two MAPK genes (*MPK3*) were also up-regulated by *Fg* and expressed higher in Shaw than in the resistant genotypes.

### Chromosome distribution of wheat DEG groups

The patterns of gene frequency distribution on wheat chromosomes among the DEGs and those putatively associated with FHB susceptibility were similar to the global gene distributions (Additional file 8). In contrast, among the various groups putatively associated with FHB resistance, the DEGs were more clustered. For example, between Nyubai and HC374, there were more common genes on chromosomes 6 (A, B, D), including two groups of homolog genes. The 107 genes specific to Wuhan1 were mostly from chromosomes 2, 4 and 5 (A, B, D), including 4 pairs of homolog genes on chromosomes 4; while the 50 genes specific to Nyubai were mostly from chromosomes 2 and 3 and included very few homologs. Although there were only 21 genes specific to HC374, 10 of them were located on chromosomes 6, including 9 acid beta-fructofuranosidase genes.

### Transcriptomics of the pathogen – *F. graminearum*

Expression profiles of pathogen genes were quite uniform. The highest number of DEGs appeared in the susceptible Shaw at 4 dpi (Fig. 2b, Additional file 9), which was consistent with the abundance of pathogen mRNA reads, pathogen biomass estimated by *GAPDH* expression, and DON concentration (Fig. 1). There were very few pathogen genes that were preferentially expressed in any of the FHB resistant hosts, but over half (4443) of the DEGs were significantly (*p* < 0.05) preferentially expressed in the susceptible Shaw. Over 8805 pathogen genes showed differential expression between the host plants, especially between a resistant wheat genotype and the susceptible Shaw. There was a significant number of genes, over 50% at 2 dpi and 96% at 4 dpi, that were more highly expressed in the susceptible Shaw than in any of the resistant host plants, but no single gene was expressed significantly higher in a resistant plant than in Shaw (Table 10). A significant number of genes (4350) expressed higher in Wuhan 1 than in Nyubai at 4 dpi, consistent with the proportion of *Fg* reads in the two genotypes (Fig. 1a, Additional file 9C). Among the 8811 differentially expressed pathogen genes, 8802 were in one major cluster that was significantly correlated (*p* < 0.05) with the abundance of pathogen mRNA read (*R* = 0.95), pathogen biomass estimated by *Fg*-*GAPDH* (*R* = 0.87), and DON concentration (*R* = 0.92) (Additional file 9D).

**Table 10:** Number of *Fg* genes expressed differentially between the host plants

	W/N_2d	W/N_4d	HC/S_2d	HC/S_4d	N/S_2d	N/S_4d	W/S_2d	W/S_4d
Up	0	4350	0	0	0	0	0	0
Down	20	0	4661	8785	4771	8794	4873	8537

**Fig. 14:**
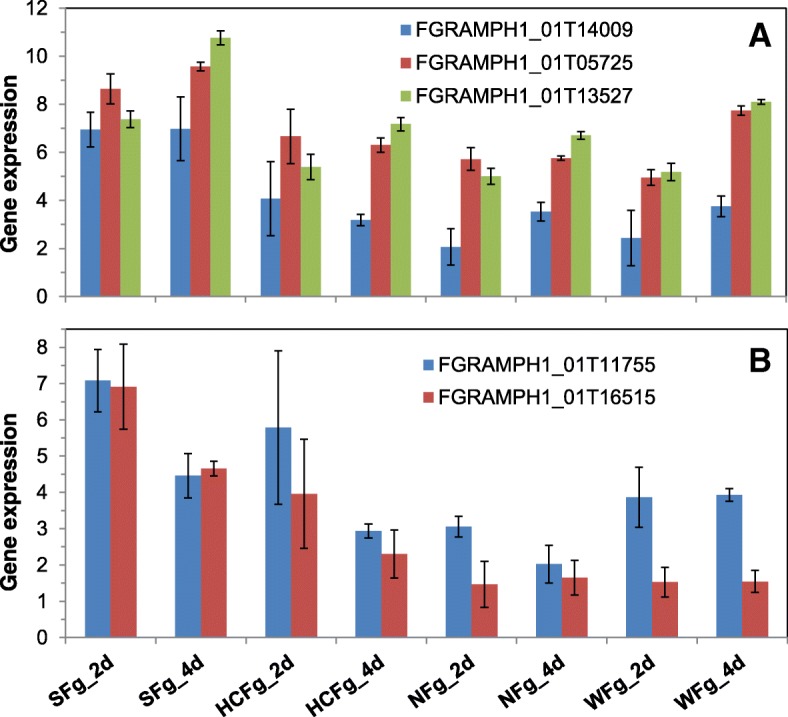
Expression profiles of five *Fg* pectate lyase genes. **a** in expression cluster C1; **b** in expression cluster C2. Fg: treatment with *Fg*; 2d and 4d: 2 and 4 dpi; S: Shaw; HC: HC374; N: Nyubai; W: Wuhan 1

Over half of the DEGs were consistent at both time points and 43% were consistent at 4 dpi across all three FHB resistant genotypes (FRS222222 = 4498, FRS020202 = 3774). There were only two genes having the DEFE pattern of FRS202020, expressed significantly higher at 2 dpi in Shaw than in all three resistant genotypes; one is a pectate lyase (FGRAMPH1_01T16515), and the other is a hydrolase (FGRAMPH1_01T12653). There were five pectate lyases among the 8811 pathogen DEGs, one (FGRAMPH1_01T11755) had very similar DEFE pattern (FRS002020) and was in the same small C2 cluster as FGRAMPH1_01T16515 (Fig. 14b). The other three pectate lyase genes were in the C1 cluster (Fig. 14a). There are totally seven pectate lyase genes in the entire *Fg* genome.

The DEFE patterns FRS020202 and FT2000 indicate that differential expression between the susceptible Shaw and all three resistant genotypes at 4 dpi and the difference between the two time points were significant only in Shaw. Joining these patterns FRS020202∩FT2000 resulted in 2431 DEGs. The GO terms described in Additional file 2G for these DEGs were enriched with genes involved in regulation of transcription (GO:0003700, *p* < 5E-19; GO:0006355, *p* < 3E-9), aromatic compound metabolism (GO:0006725, *p* < 4E-07), and regulation of nitrogen compound metabolism (GO:0051171, *p* < 6E-07). An enrichment in acetyltransferase activity (GO:0016407, *p* < 0.002), mainly N-acetyltransferase activity (GO:0008080, *p* < 0.003) was also noted.

## Discussion

In our dataset, the higher number of DEGs of both host and pathogen in the susceptible Shaw, especially at 4 dpi, was closely associated with higher *Fg* biomass and extent of infection. A similar phenomenon was noticed in another study on different genotypes (Wang L, Li Q, Liu Z, Surendra A, Pan Y, Li Y, et al: Integrated transcriptome and hormone profiling highlight the role of multiple phytohormone pathways in wheat resistance against fusarium head blight, submitted). Our analyses showed that distinct groups of genes were activated at different stages in the individual genotypes in response to *Fg* infection. This is evident in this study among both groups of genes associated with FHB resistance and susceptibility.

### Genes associated with *Fg* challenge

About 40% of the DEGs in this dataset were up-regulated after *Fg* treatment, and the large majority were up-regulated in all four genotypes investigated, with the up-regulation in Shaw being the largest. Gene ontology analysis showed up-regulation of a number of specific protein kinases and transcription factors, suggesting reprogramming of gene regulatory pathways. Up-regulation of such molecular functions was observed in other wheat-*Fg* transcriptomic studies and with treatment of *Fg* mycotoxin DON [16–18]. We also observed up-regulation of key processes associated with biotic stress such as regulation of cell death, response to unfolded protein and regulation of immune response. The response to unfolded proteins is activated when the endoplasmic reticulum becomes overwhelmed by the cell’s biosynthetic demands and accumulates unfolded or misfolded proteins; it can lead to the initiation of cell death when the situation remains out of control [19]. Cell death also plays an important role in the immune response in plants [20]. Our analysis suggests that the intensity of the wheat response to *Fg* infection, associated with a massive reprogramming of gene transcription, overwhelmed the endoplasmic reticulum and may have triggered apoptosis, especially in the susceptible genotype. In addition, many up-regulated plant defense regulatory genes were also correlated with the regulation of cell death. Although cell death is a successful strategy to stop a biotrophic fungus, it may contribute to the success of a hemi-biotrophic fungus such as *Fg*.

Nitronate monooxygenases (EC1.13.12.16, NMO) are essential to mitigate nitro-oxidative cellular damage and contribute to the maintenance of redox balance in stress conditions [21]. In the fungus *Magnaporthe oryzae*, expression of this enzyme is associated with self-detoxification as well as suppression of the rice immune response. NMOs are known to oxidize alkyl nitronates to aldehydes and nitrite in plants and fungi, and to detoxify the metabolic inhibitor propionate-3-nitronate, toxic to all organisms that use succinate dehydrogenase [22, 23]. NMO is also found to be associated with multiple herbicide resistance in *Lolium multiflorum* [24]. The up-regulation of the wheat NMOs correlating with the level of *Fg* infection*,* in this study, suggests a potential role in the immune response of wheat.

Significant increases in aromatic amino acid metabolism, tryptophan biosynthesis in particular, and nitrogen compound metabolic process were also noted. Tryptophan is a precursor for many phenylpropanoids and for lignin; these contribute to strengthening of the primary cell wall, and are thought to participate in blocking of fungal progression in plant tissues (reviewed in [25]). Many studies have noted an up-regulation of tryptophan and phenylpropanoid biosynthesis pathways after *Fg* infection [17, 26–28].

About 21% of the DEGs were down-regulated by *Fg* treatment, and the majority was down-regulated to a larger extent in Shaw, especially at 4 dpi. There was an important effect on microtubule-based processes and on primary metabolic processes, including nitrogen compound metabolism, glucan biosynthesis, chlorophyll biosynthesis and photosynthesis. Processes associated with DNA repair and replication, gene silencing by RNA, translation and protein folding were also negatively affected. Down-regulation of primary metabolism and photosynthesis has been reported in different plant-pathogen interactions and has been suggested to be a mechanism to alleviate the energy costs associated with the up-regulation of other metabolic pathways for defense [5, 6]. Down-regulation of ribosomal proteins in an FHB-susceptible genotype by *Fg* and DON has been reported by Foroud et al. [26]. DON is known to inhibit protein synthesis. A negative effect of *Fg* infection on microtubule-based processes, DNA repair and replication, and protein folding has not, to our knowledge, been reported before. Microtubules-based processes contribute to many cellular activities, including intracellular transport, secretion, cell structure, and chromosome separation. It has been shown that disruption of microtubule networks suppresses cell wall-mediated defense in *Arabidopsis thaliana* [29]. Heat shock proteins are key components of protein folding; many of them have been reported to have a positive role in regulation of plant immunity [30]. The down-regulation of genes associated with protein folding in our experiments correlated with the up-regulation of the response to unfolded proteins.

Overall, the response of wheat to *Fg* infection included a large reprogramming of cellular processes which were mostly shared between susceptible and resistant genotypes, although with a larger magnitude in the susceptible genotype. Wheat invested a lot of energy, both in susceptible and resistant genotypes, towards extensive transcriptional reprogramming. That effort, particularly in the susceptible genotype, appeared to be insufficient to mount an effective plant defense against FHB.

### Differential gene expression associated with *Fg* treatment and susceptibility to FHB

In the susceptible Shaw, 543 wheat genes were uniquely up-regulated at 2 dpi and 2204 at 4 dpi; among these genes, 477 were detected at both 2 and 4 dpi. More than 30% of these genes were part of the gene association network, supporting a coordinated reprogramming of gene transcription. The earlier and higher differential expression of many genes in susceptible, FHB-challenged plants may be attributable to earlier and higher levels of *Fg* infection, triggering activation of plant defense and other responses. Although “plant susceptibility genes” have been documented such as *PMR6* required for susceptibility to powdery mildew in *Arabidopsis* [31], the larger extent of genes with differential expression in susceptible, infected plants do not fall in this class.

A subgroup of *LRR-RKs* was up-regulated early (2 dpi) in Shaw and not in the resistant genotypes, followed by up-regulation of many other types of kinases and of many types of transcription factors at 4 dpi. Additional kinases and transcription factors were up-regulated only in Shaw at both time points (Table 7). LRR-RKs play important roles in plant immunity, by recognizing pathogen-associated molecular patterns (PAMPs) associated either with the pathogens or the attacked host and processing the extracellular signals through signaling cascades and defense interaction networks [31–34]. Specific LRR-RKs have been associated with resistance to specific pathogens in many crops, including in wheat (e.g. powdery mildew, [35]). However, the blocking or diverting of LRR-RK signaling by a pathogen has also been reported [31, 36]. Given the large transcription reprogramming happening in the susceptible genotype, one may ask if *Fg* can hijack wheat signaling. *WRKY*, *Myb* and ET-responsive transcription factors have been shown to be part of signaling cascades activated by LRR-RKs [9, 37, 38]. Very few LRR-RKs and associated networks have been characterized in wheat. So it is yet too early to confirm whether the *LRR-RKs*, transcription factors and kinases up-regulated only in Shaw in early infection are part of defense or other types of responses.

The ubiquitin/proteasome system has been shown to play an important role in regulating the plant immune response, including by degradation of receptors activating the immune response [39, 40]. In Shaw, we observed the up-regulation of many F-box proteins, used by the ubiquitin/proteasome system to recognize proteins targeted for degradation. Identification of the targets for those F-box proteins will be required to determine if they contribute to the FHB susceptibility in Shaw.

### Differential gene expression associated with *Fg* treatment and resistance against FHB

#### DEGs common to the three resistant genotypes

The 12 DEGs associated with FHB resistance that were up-regulated in all three FHB-resistant genotypes examined in this study were enriched in functions associated with early defense response in plants. Four kinase proteins, two *LRR-RKs* and two with lectin domains, were strongly up-regulated in the FHB-resistant genotypes. Kinases of this type are referred to as pattern recognition receptors (PRRs). Upon recognition of specific PAMPs by PRRs, the PAMP-triggered immunity response is activated, leading to activation of MAP kinases, ROS production and transcriptional reprogramming including up-regulation of *MAPK*, *WRKY*, *MYB* and ET-responsive transcription factors [41–43]. A large number of predicted PRRs are present in wheat; however very few have been characterized. One or more of the kinases identified here could contribute to the perception of *Fg* in the FHB-resistant genotypes.

The three glutathione S-transferases (*GSTs*) were part of a group of 261 *GSTs,* which were differentially expressed in the analyzed dataset, and only one of the three was part of an association network. Modulation of the redox state of glutathione by GSTs regulates early signaling events in biotic stresses such as fungal infections, including activation of the essential regulator of systemic acquired resistance NPR1 [44]. In the *npr1–3* mutant of *Arabidopsis thaliana*, the expression of *GST* (At2g47730) and *SOBIR1* (At2g31880) is severely impaired as compared to the wild type when subjected to SA perturbation [45]. GSTs are known for their roles in maintaining the physiological redox state of the cell, protection of the cell against oxidative damages and detoxification of xenobiotics [46, 47]. Although up-regulation of *GSTs* has been observed in FHB-resistant as well as FHB-susceptible wheat material following *Fg* infection, increased levels of specific *GST* family members has been observed in wheat and barley in association with FHB resistance [26, 27, 48, 49].

Two components of the phosphatidylinositol signaling pathway were expressed at higher level in the three FHB-resistant genotypes, a phosphatidylinositol 4-phosphate 5-kinase (*PIP5K*, EC:2.7.1.68) and a hyccin-like gene. Hyccin is part of a complex that localizes phosphatidylinositol 4-kinase to the plasma membrane and stabilizes its activity [50]. In *Arabidopsis* cells, phosphatidylinositol 4-kinase and *PIP5K*, involved in two consecutive steps in phosphatidylinositol phosphorylation, are both activated within minutes by SA treatment [51]. Phosphatidylinositol 4-kinase has been shown to be part of a complex that negatively regulates SA-dependent gene expression and defense response [52]. The higher expression of hyccin and *PIP5K* in FHB-resistant material could be interpreted as a tone-down of the SA-dependent defense response in the FHB resistant genotypes. This is consistent with our analysis of DEGs associated with hormone pathways (see below).

Purple acid phosphatases (PAPs, EC 3.1.3.2), which was up-regulated in the three FHB resistant genotypes but not in Shaw, have important roles in intracellular and extracellular phosphorus scavenging and recycling, including P-remobilization during tissue senescence, however their physiological roles are poorly understood [53]. Recently, the purple acid phosphatase PAP5 from *A. thaliana* has been shown to be required for maintenance of basal resistance and to work upstream of SA accumulation [54]. Further work will be required to understand their contribution to defense response in wheat.

The MatE transmembrane transporter is part of the large family of multidrug and toxic compound extrusion (MATE) proteins that are known to transport secondary metabolites and xenotic compounds. Although only a few of those transporters have a characterized function, some have been identified as key players in stress and pathogen response. For example, the rice MATE1 and MATE2 and *Arabidopsis* ADS1 are negative regulators of disease resistance [55, 56] while *Arabidopsis* EDS5 is associated with disease tolerance via a SA pathway [57]. The MatE gene TraesCS2B01G296000 identified in this study as associated with resistance is not homologous to any of the characterized MATE proteins.

#### Other DEGs associated with resistance

We identified a higher number of DEGs with similar expression profiles between HC374 and Nyubai than in the other two comparisons, both in correlation analysis and in the DEFE analysis. This suggests that the progeny line HC374, resulting from a cross between Nyubai and Wuhan 1, is genetically closer to Nyubai than Wuhan 1 in terms of resistance to FHB. This is consistent with the fact that HC374 and Nyubai share two QTLs on chromosomes 3BS and 5A, while HC374 and Wuhan 1 share one weaker QTL on chromosome 2DL [4].

Among the 17 DEGs strongly up-regulated by *Fg* in both Nyubai and HC374, there were four *LRR* containing proteins (including TraesCSU01G181100), two with transmembrane domains and possible receptor activity as PRR, and two located either in the cytoplasm or nucleus that could play a role in elicitor-triggered immunity (ETI). Notably, the receptor protein kinase TraesCS6A01G417900 is orthologous to the *Arabidopsis* chitin elicitor receptor kinase *CERK1*. This kinase has been shown to be required for the non-host defense response of *Arabidopsis* to *Fusarium oxysporum* [58]. The barley ortholog *HvCERK1* has been shown to be up-regulated by *Fg* and to contribute to FHB resistance [59]. Transient silencing of *HvCERK1* is also associated with lower expression of a *MAPK* and two *WRKY* genes as well as lower production of phenylpropanoid and flavonoid metabolites. The up-regulation of phenylalanine ammonia lyase, a key enzyme in the biosynthesis of phenylpropanoids, in this group of genes common between Nyubai and HC374 is consistent with the possibility that TraesCS6A01G417900 has *CERK1*-like activity in wheat and may contribute to resistance to FHB in the two genotypes. Also in common between Nyubai and HC374 were genes associated with secondary metabolism, including an acetylglucosaminyltransferase, a key enzyme for the biosynthesis of terpenes, sesquiterpene synthase, and other genes associated with stress response and detoxification (*OMA1*, peroxidase, *MatE* transporter). Increased expression of biosynthesis genes for phenylpropanoid and terpenoid compounds has been observed in wheat carrying the *Fhb1* gene for FHB resistance, located on the chromosome 3BS [60]. This gene is also carried by both Nyubai and HC374.

The group of 50 DEGs unique to Nyubai contains an additional 15 kinases and receptor-like kinases including many that were up-regulated at much higher levels at 2 and 4 dpi in Nyubai in response to *Fg* infection. The increased expression of multiple kinases has been associated with FHB resistance in other wheat material [27, 48, 49]. Up-regulation of numerous kinases suggests an effort (or an attempt) to modulate gene expression. However, higher up-regulation of known gene sets associated with defense and detoxification was not observed in the time frame analyzed. In contrast, the 107 DEGs unique to Wuhan 1 contained a large number of defense related genes that were transiently up-regulated at a higher level at 2 dpi in that genotype compared to the other genotypes. These observations were supported by network analysis and suggest the use of different defense strategies by these resistant parents.

Only a small number of genes unique to the hybrid HC374 were up-regulated relative Nyubai and Wuhan 1; the close genetic relationship with its parental genotypes explains this at least in part. Surprisingly, 9 of the 12 differentially expressed acid beta-fructofuranosidases, or acid invertases, in our dataset had a much higher up-regulation in HC374 at 4 dpi. Cell wall invertases have been shown to be essential for proper sugar partitioning and signaling to induce defense response during pathogen attack [61].

Eight DEGs with similar strong up-regulation of expression in Wuhan 1 and Nyubai, but not in HC374 were enriched in genes with secondary metabolic transfer activity (transferases, cytochrome P450, blue copper proteins). This could be an attempt by both genotypes either to detoxify xenobiotics or to produce/modify secondary metabolites. The observation that most of the DEGs were expressed at much lower level in HC374 suggests that they are not essential to FHB resistance. However, up-regulation of glycosyltransferases and P450s has been observed in FHB-resistant barley and wheat, including Chevron, CM82036, Dream, Nobeokabouzu and Sumai 3 [27, 48, 62, 63].

### Genes associated with hormonal pathways and pathogenesis-related proteins in response to FHB challenge

There are contradictory reports in the literature related to hormonal signaling pathways and their contribution to FHB resistance in wheat. Makandar et al. [64, 65] showed that SA-induced expression of *PR1* was associated with FHB resistance. In contrast, many studies have observed an increase in JA level and pathway activity in FHB resistant genotypes [16, 25, 62]. There have been suggestions that the regulation of pathogenesis-related genes by plant hormones is genotype-dependent in wheat [66], and evidence that the timing of activation of the SA and JA pathways is critical to determine resistance or susceptibility to *Fg* in *Arabidopsis* [67]. Observations in wheat supported this finding [68]. In addition, ET signaling is shown to facilitate *Fg* infection in *Arabidopsis* and wheat [69].

In the genotypes characterized here, the majority of the DEGs associated with ET and JA pathways showed a stronger up-regulation by *Fg* in the susceptible Shaw. This included the positive regulators of ET response ethylene insensitive 3 and *MBF1C*. MBF1C is a transcriptional coactivator that modulates ET-response signal transduction. Overexpression of *MBF1C* gene in *Arabidopsis thaliana* has been shown to confer enhanced tolerance to bacterial infection, heat and osmotic stresses [70]. Genes for at least three steps in the biosynthesis of JA, allene oxide synthase and cyclase, and 12-oxophytodienoate reductase, were more strongly up-regulated in Shaw than in the 3 resistant genotypes. Many lipoxygenases, involved in the biosynthesis of JA and other oxylipins, were also up-regulated by *Fg*. Nalam et al. [71] have shown that inactivation of the lipoxygenase *TaLpx-1*, essential to oxylipin synthesis, resulted in enhanced resistance against *Fg* in wheat; they also showed that inactivation of the ortholog gene *LOX1* in *Arabidopsis* lead to a stronger SA pathway activity and attenuation of JA signaling during *Fg* infection.

There was also up-regulation of many JA *ZIM* domain protein genes, including 5 associated with FHB susceptibility; these are repressor of JA signaling. However, conjugation of JA to isoleucine by amido synthetases, including indole-3-acetic acid-amido synthetase GH3, triggers the degradation of the JA ZIM domain proteins and induces signaling [72]. Given that many indole-3-acetic acid-amido synthetase *GH3.3* were up-regulated in the genotypes characterized, it is possible that JA-isoleucine conjugation occurred. Up-regulation of *PR4* and thaumatin genes by *Fg* is suggestive of JA signaling activity.

There was a much smaller number of DEGs up-regulated by *Fg* associated with the SA pathway. These included DEGs annotated as *NPR1*, *NRR* repressor homolog 1, salicylate o-methyltransferase, accelerated cell death 11 (*ACD11*), phytoalexin-deficient 4 (*PAD 4*) and *MPK3.* In *Arabidopsis*, these genes contribute to the regulation of SA levels and signaling pathways. Salicylate o-methyltransferase regulates SA homeostasis. NPR1 is a positive transcriptional co-regulator of SA signaling and a negative one for JA, and it is needed for *PR1* expression [73, 74]; NRR repressor 1 negatively regulates NPR1-mediated transcriptional activation [75]. ACD11 is a ceramide-1-phosphate transfer protein that modulates SA-dependent programmed cell death [76]. PAD 4 promotes SA accumulation and regulates the crosstalk between SA and JA/ET pathways [77]. MPK3 inhibit SA accumulation and repress some defense response pathways [78]. Although the function of the SA-associated DEGs suggested a complex response, the up-regulation of a large number of genes from the *PR1* family was consistent with SA signaling up-regulation; however this activity was associated more with susceptibility than resistance to FHB, as suggested by the 5 *PR1* genes up-regulated only in Shaw during the time period examined.

Auxin signaling is a key component of growth and development in healthy plants. However contradictory reports exist in the literature about its role in disease. In *Arabidopsis*, auxin signaling has been reported both to promote disease susceptibility to bacterial infection and contribute to resistance to necrotrophic fungi [79, 80]. In rice, *SAUR*-like genes have been shown to negatively regulate auxin synthesis and transport [81]; suppression of auxin activity promotes basal immunity [82]. The role of auxin in the response to *Fg* infection in wheat is poorly characterized. Biselli et al. [17] observed that genes associated with auxin metabolism and signaling were induced by FHB in both the susceptible and resistant wheat genotypes that they characterized, but at a higher level in the susceptible one. We observed that many genes from two families associated with negative regulation of auxin activity, the *SAUR*-like and *GH3.3* genes were strongly up-regulated by *Fg* infection, especially in Shaw. GH3.3 can conjugate auxin (in addition to JA) to amino acids, contributing to auxin homeostasis by inactivation [82]. Auxin efflux carrier proteins and influx transporters were respectively up- and down-regulated by *Fg* infection; these are involved in regulation of auxin homeostasis and their differential expression profiles suggest an attempt to move or keep auxin out of cells. In addition, many of the auxin-response factors and auxin-responsive protein genes were down-regulated by *Fg* infection, consistent with a negative regulation of auxin activity. Qi et al. [83] showed that large amounts of auxin were detected at 4 dpi with *Fg* in a susceptible wheat genotype, possibly produced by the fungus. *Fg* may use auxin as a susceptibility factor and the up-regulation of *SAUR*-like, *GH3.3* and efflux carrier protein genes may be an attempt by wheat to deal with the excess of auxin.

The expression profiles of genes associated with the ABA signaling pathway suggested activity towards reduction of ABA signaling, especially in the susceptible Shaw. The up-regulation by *Fg* of the two RING/U-box superfamily protein genes, orthologous to *Arabidopsis AIP2* – a negative regulator of ABA signaling, and the five cytochrome P450 family ABA 8′-hydroxylase genes, homologous to *Arabidopsis CYP707A* family – key players in the regulation of ABA levels via catabolism [84], supports that interpretation. Surprisingly, the three molybdenum cofactor sulfurase genes (*ABA3*), known as key positive regulators of ABA biosynthesis, were also significantly up-regulated by *Fg*. Overexpression of *ABA3* has been reported to be associated with enhanced abiotic stress tolerance in *Arabidopsis*, maize and rice [85, 86]. Our dataset suggests an association of ABA activity with FHB susceptibility. It has been shown that exogenous treatment of wheat heads with ABA accelerates apparition of disease symptoms in a susceptible wheat cultivar [83].

Overall, our analysis of hormonal pathways indicates that increased gene expression activity in the JA, ET, SA, auxin and ABA pathways were associated with FHB susceptibility in the genotypes characterized. The number of DEGs associated with FHB susceptibility, especially from the auxin, ET and JA pathways, as well as the expression profile of many pathogenesis-related genes up-regulated by *Fg* treatment supports this association. The large number of up-regulated genes in hormone pathways and pathogenesis-related gene families illustrates the large effort made by wheat to mount a defense response that is either not sufficient or not productive in the genotypes characterized.

### Transcriptomics of the pathogen

Of the five pectate lyase genes highly expressed in Shaw, the two expressed at higher levels at 2 dpi may contribute to initial penetration of the plant host cell wall rather than propagation of infection within the host. Plant pathogenic fungi secreted cell-wall degrading enzymes such as pectate lyases are important in degradation of plant pectin, a polymer of galacturonic acid found in the middle lamella of the cell wall. Pectinases are the first enzymes to be secreted by fungal pathogens when they attack plant cell walls [87, 88]. Inactivation of a pectate lyase in *Fusarium solani* reduced the ability of the fungus to infect plant tissues [89].

Significant enrichment of pathogen acetyltransferase expression after infection was evident in this study, especially in the susceptible host Shaw at 4 dpi. It is well known that plant immune response during pathogen infection requires extensive transcriptional reprogramming involving histone acetylation. Pathogens interfere with this process by using effector proteins encoding acetyltransferases that can directly acetylate host proteins to alter immunity [90, 91].

We found very few *Fg* genes up-regulated at a higher level in resistant genotypes as compared with the susceptible one at 2 and 4 dpi. This is in contrast with the findings of Hofstad et al. [92], who found 112 genes expressed at a higher level in their FHB resistant genotype at 4 dpi. Fungal strain or host genotype differences may explain this difference.

## Conclusions

This study explored global transcriptomic profiles of four different wheat genotypes treated with *Fg*. In both the resistant and susceptible genotypes, transcriptional reprogramming was evident upon FHB challenge. In the susceptible genotype Shaw, a sub-group of *LRR-RKs* was uniquely up-regulated by *Fg*, followed by up-regulation of many transcription factors and numerous genes associated with defense response. However, this activity was not sufficient to prevent the spread of *Fg* infection. Differential gene expression patterns associated with the resistant genotypes, including patterns common to two or three of them and patterns unique to individual resistant genotypes, indicated multi-facetted defense responses that were distinct for each resistant genotype.

## Methods

### Plant material and RNA extraction and sequencing

Four wheat (*Triticum aestivum*) genotypes (i.e. cultivars or lines) were used in this study: Wuhan 1 (Type II FHB resistant), Nyubai (Type I FHB resistant), HC374 (a FHB resistant double haploid line derived from crossing Wuhan 1 with Nyubai), and Shaw (a FHB susceptible cultivar). Seeds from the three resistant genotypes were kindly provided by Dr. George Fedak (Ottawa Research and Development Centre, Agriculture and Agri-Food Canada). Somers et al. [4] identified 4 QTLs for FHB resistance located on chromosomes 2DL (carried by Wuhan 1 and HC374), 3BS (Nyubai and HC374), 4B (Wuhan 1) and 5A (Nyubai and HC374).

Wheat plants were grown in controlled-environment cabinets with 16 h light at 20 °C and 8 h dark at 16 °C until mid-anthesis then transferred to growth chambers at anthesis. Heads at mid-anthesis were point inoculated with either water (control), or *Fg* (strain DAOM233423). Plant growth, inoculation and infection conditions were described in detail previously [93]. Inoculated spikelet samples were collected in triplicate (from 5 heads per replicate) at 2 and 4 days post inoculation (dpi). In total, 48 samples were collected; total RNA, containing both the plant and pathogen RNA present in the samples, was extracted using the TriReagent (Molecular Research Center Inc.) followed by a treatment with DNase I (RNase-Free DNase set, Qiagen) onto columns (RNeasy Mini kit, Quiagen) according to instructions provided by the manufacturer. RNA was processed for deep paired-end RNA sequencing using Illumina HiSeq 2500 by the National Research Council Canada sequencing service.

### RNA-seq data processing

The International Wheat Genome Sequencing Consortium (IWGSC) RefSeq v1.0 complete reference genome and corresponding annotation v1.0 [94] were used as reference for the analysis of wheat RNA-seq data. Following recommendations of IWGSC, the chromosome-partitioned version of the Chinese Spring version 1.0 genome was used and the gff3 file was reformatted accordingly. IWGSC provided both high and low confidence gene models for options; in this study, only the high confidence gene models were used. *Fusarium graminearum* reference genome (Fusarium graminearum *str. PH-1*) was obtained from EnsemblFungi [95]. Wheat and *Fg* genomes were combined into a host-pathogen pan-genome, and annotation data from both species were combined into a pan-annotation (Liu Z, Li Y, Pan Y, Wang L, Ouellet T, Fobert P: Strategy in wheat-Fusarium dual genome RNA-seq data processing, in preparation). This pan-genome contains 124,935 gene models, 14,145 from *Fg* and 110,790 from wheat. The RNA-seq reads were preprocessed by trimming adaptor sequences, filtering low-quality reads (Phred Score < =20 [96]) and eliminating short reads (length < = 20 bps) using FASTX Toolkit [97, 98]. After filtering, barcode and adaptor removal, an average of 24.5 million paired reads per sample were retained for subsequent read mapping through the RNA-seq data processing procedures. The cleaned RNA-seq reads were mapped against the pan-genome using STAR v2.5.3a [99] to generate gene-level counts. DESeq2 [100] was used for data normalization and subsequent DEG analysis for each pairwise comparison. Normalized read counts along with log2 fold change and *p*-values were used for downstream data analysis.

### Data reduction and feature pattern identification

Absolute value of log2 fold change > = 1 and *p*-values <=0.01 were applied to filter all datasets. For differential analysis, we retained only genes with a minimum of 100 reads in at least one of the samples compared. We further applied the Differential Expression Feature Extraction method (DEFE, Pan Y, Li Y, Liu Z, Surendra A, Wang L, Foroud NA, Goyal RK, Ouellet T, Fobert PR: Differential expression feature extraction and its application in wheat RNA-seq data analysis, forthcoming) to identify differentially expressed genes. Gene expression data presented in the result section are log2 transformed using a pseudo-count of +1.

### Gene ontology enrichment analysis and orthology

Gene ontology (GO) annotations were compiled by combining three versions of *Triticum aestivum*: (1) IWGSC RefSeq v1.0 annotation from [94], (2) Ensembl Plants TGACv1 (from Ensembl Plants release 34) and (3) IWGSC survey genome 2.2 (from Ensembl Plants release 28 [101]). The mapping of gene IDs between the three versions was based on the tables obtained from URGI (between IWGSC RefSeq v1.0 and Ensembl Plants TGACv1) and Ensembl Plants (between Ensembl Plants TGACv1 and IWGSC survey genome 2.2). The resulting GO annotations are available in Additional file 10. Gene ontology enrichment analysis was conducted using Gene Ontology Analyzer [102].

Orthologs in *Arabidopsis thaliana* (Araport11, from TAIR: [103]) and *Brachypodium distachyon* (Bdistachyon_314, from JGI: [104]) were obtained for *Triticum aestivum* genes. One-to-one reciprocal best hit (RBH) was performed between each of the *Triticum aestivum* sub-genome A, B and D, respectively, on one side and the Araport11 or Bdistachyon_314 on the other.

### Association network and pathway analysis

Gene association network analysis of the differentially expressed gene dataset was conducted using the WGCNA R package [105]. A Pearson correlation coefficient matrix *C* was first computed, and then the positive adjacency matrix and negative adjacency matrix were computed, respectively: *A*_+_ = (*C*_+_)^*e*^, *A*_−_ = (| *C*_−_| )^*e*^, where *C*_+_ and *C*_−_ are the positive and negative correlation coefficient matrices and *e* is the power for soft-thresholding. Based on these adjacency matrices, a TOM similarity matrix was generated for each adjacency matrix, and the two TOM matrices were merged into a similarity matrix using:

1 $$ S=\frac{Tomsimilarity\left({A}_{+}\right)+ Tomsimilarity\left({A}_{-}\right)}{2} $$

Gene association network analysis was performed using the similarity matrix (***S*** in Eq. 1) as edge weights. The top 1% weight in ***S*** was considered as valid edges in the network.

Hierarchical clustering was employed based on the similarity matrix (1-*S* as distance measure) to cluster genes. Dynamic tree cutting was used to generate gene clusters/modules using the dynamicTreeCut R package [105]. We digitalized the experimental traits (Fg treatment, time post inoculation) as described in [106]. The five FHB-related traits (Fg treatment, Fg_time, %Fg, Fg_GAPDH, and DON, Fig. 6, Additional file 1C) were used for Pearson correlation analysis between each of them on one side and expression profile of each gene or centroid (eigen gene resulted from WGCNA) of each cluster on the other side. Results of these correlation analyses are presented in Additional file 1A (Cols BD-BE) and Additional file 1D.

An enrichment index was defined to describe the extent of enrichment of certain group of genes among a subset of the wheat genome, such as the DEGs dataset or a set of network nodes. The global abundance, *g* is defined by the proportion of this group of genes in the wheat genome, which is the global frequency of this group of genes, *f*, divided by the size of the wheat genome, *G*: *g* = *f*/*G*. Similarly, the abundance (*d*) of this group of genes in the subset of the genome (*D*) is defined by its proportion in the subset of the genome, which is the frequency (*f*_*d*_) of this group of genes in the subset *f*_*d*_: *d* = *f*_*d*_*/D*. An enrichment index (*E*) is defined as *E* = *d*/*g*.

### RT-qPCR validation

The cDNA synthesis of all RNA samples was carried out with the RETROscript® reverse transcription kit (Fisher Scientific, Waltham, MA, USA), using 2 μg of each RNA sample into a 20-μl reaction volume with oligo(dT)18 primer, and all manipulations followed the manufacturer’s protocol. SensiFast SYBR No-Rox kit (Bioline) and the MJ Research PTC200 thermal Cycler with Chromo 4 detector were utilized to performed RT-qPCRs, with 10 min at 95 °C, 35 cycles of 30 s at 95 °C, 30 s at melting temperature, and 1 min at 72 °C, melting curve from 55 °C to 95 °C, read every 1 °C, hold 5 s. Two technical replicates were done for each of the three biological replicates. Genes selected for RT-qPCR analysis respected two of three criteria: 1) being expressed at a sufficiently high level to be detectable by RT-qPCR analysis; 2) having sufficient long region(s) of unique sequence, allowing the design of genome-specific primers; or 3) having a closely related expression profile to their homologous genes from wheat sub-genomes A and B. Primers used are described in Additional file 11. Fungal biomass was estimated using expressed levels of *GAPDH* (*FGSG_06257*). The 2^-ΔΔCt^ method [107] was used to calculate FC values, and the relative expression levels were normalized against three wheat reference genes, *IAAOx* (TraesCS2A01G246300), *AOx* (TraesCS2A01G327600), and *hn-RNP-Q* (TraesCS2A01G390200), as calculated by Vandesompele et al. [108], and rescaled using the lowest value as 1 among compared samples.

## Additional files

Additional file 1: The 28,961 differentially expressed wheat genes. This file also includes (B) statistics of differential expression feature extraction (DEFE) patterns associated with these genes, (C) values of FHB-related traits, (D) Pearson correlation between centroids (eigenvalues) of the clusters and the FHB-related traits. (XLSX 19261 kb)

Additional file 2: Results of GO enrichment analyses. (XLSX 1033 kb)

Additional file 3: FHB resistant genes across all three resistant genotypes (A), common between two genotypes (B), and unique to individual genotypes (C). (XLSX 172 kb)

Additional file 4: Enrichment indices of gene groups in various gene populations: DEGs, FHB-resistant, FHB-susceptible, up- and down-regulated by *Fg*, networks, key hub genes. (XLSX 2244 kb)

Additional file 5: Annotation of the six genes currently annotated to encode erect panicle 2 protein need to be updated. Two groups of genes with annotation of “Erect panicle 2 protein” in the “Human-Readable-Description” provided with IWGSC RefSeq v1.0. Annotation of these six genes needs to be revisited. (PDF 228 kb)

Additional file 6: The 57 Pathogenesis-Related genes. (XLSX 45 kb)

Additional file 7: The 542 phytohormone pathway genes. (XLSX 419 kb)

Additional file 8: The chromosomal distribution of various gene populations. (XLSX 31 kb)

Additional file 9: The 8811 differentially expressed pathogen genes. This file also include (B) statistics of differential expression feature extraction (DEFE) pattern associated with each gene, (C) values of FHB-related traits, and (D) Pearson correlation between centroids of the clusters and the FHB-related traits. (XLSX 5432 kb)

Additional file 10: Gene ontology annotations compiled from three versions of the wheat genome: IWGSC Survey genome 2.2, TGACv1 and IWGSC-RefSeqv1.0. (XLSX 18572 kb)

Additional file 11: Primers used for RT-qPCR analyses in this study. (XLSX 13 kb)

## Abbreviations

ABA: Abscisic acid
AOx: Amine oxidase
AP2/ERF: APETALA2/ethylene-responsive transcription factor
bHLH: basic helix-loop-helix domain containing transcription factor
bZIP: basic leucine zipper domain containing transcription factor
C_t_: Threshold cycle
DAMP/PAMP: Damage/pathogen associated molecular pattern
DEFE: Differential expression feature extraction
DEG: Differentially expressed gene
DON: Deoxynivalenol
dpi: days post inoculation
ET: Ethylene
ETI: Elicitor-triggered immunity
FC: Fold change
*Fg*: *Fusarium graminearum*
FHB: Fusarium head blight
GAPDH: Glyceraldehyde-3-phosphate dehydrogenase
GO: Gene ontology
hn-RNP-Q: heterogeneous nuclear ribonucleoprotein Q
IAAOx: Indole-3 acetaldehyde oxidas
IWGSC: International Wheat Genome Sequencing Consortium
JA: Jasmonic acid
LOX: Lipoxygenase
LRR-RK: Leucine-rich repeat receptor kinase
MADS box: MADS box domain containing transcription factor
MAP: Mitogen-activated protein
MAPK: Mitogen-activated protein kinase
MYB: Myeloblastosis domain containing transcription factor
NAC: NAC domain containing transcription factor
NMO: Nitronate monooxygenases
NPR: Nonexpresser of PR (pathogenesis-related) genes
NRR: Negative regulator of resistance
OMA: Metalloendopeptidase
PAD: Phytoalexin-deficient
PIP5K: Phosphatidylinositol 4-phosphate 5-kinase
PR: Pathogenesis-related
PRR: Pattern recognition receptor
QTL: Quantitative trait locus
ROS: Reactive oxygen species
SA: Salicylic acid
SAUR: Small auxin-up RNA
TF: Transcription factor
TOM: Topology overlap matrix
WGCNA: Weighted Gene Correlation Network Analysis
WRKY: WRKY domain containing transcription factor
ZIM: Zinc-finger inflorescence meristem
