# Supplementary material for: Transcriptome dynamics associated with resistance and susceptibility against fusarium head blight in four wheat genotypes
Source: BMC Genomics. 2018 Aug 29;19:642. doi: 10.1186/s12864-018-5012-3 (PMC6116500; doi:10.1186/s12864-018-5012-3)
Supplement: Supplementary file 5 — Annotation of the six genes currently annotated to encode erect panicle 2 protein need to be updated. Two groups of genes with annotation of “Erect panicle 2 protein” in the “Human-Readable-Description” provided with IWGSC RefSeq v1.0. Annotation of these six genes needs to be revisited. (PDF 228 kb) [file 12864_2018_5012_MOESM5_ESM.pdf]

Additional file 5: Annotation of the six genes currently annotated to encode erect panicle 2 protein need to be updated

In the entire wheat genome, there are six genes annotated to encode erect panicle 2 protein, three from Chromosome 5 and three from Chromosome 2. The three from Chromosome 5 were in the gene association network as highly connected key hub genes (TraesCS5A01G212400, TraesCS5B01G211300, TraesCS5D01G219600) and orthologous to the rice gene named major ampullate spidroin 2 (LOC_Os09g26710), for its homology to a major spider silk protein. These three genes were drastically upregulated by *Fusarium graminearum*, especially in the susceptible Shaw (**Figure S1A**). The other three genes (TraesCS2A01G168600, TraesCS2B01G194900, TraesCS2D01G176200) from Chromosome 2 were down regulated by *F. graminearum*, more strongly in the susceptible Shaw, and orthologous to GPI-anchored adhesin-like protein (AT3G14172) and COP1-interacting protein-like protein (AT1G72410) in *Arabidopsis thaliana* (**Figure S1B**). Based on their orthologs and drastic difference in their expression profile, we believe they are two different groups of genes. This merits further investigation.

This finding has been forwarded the group working on an update of the IWGSC-RefSeq annotation for wheat.

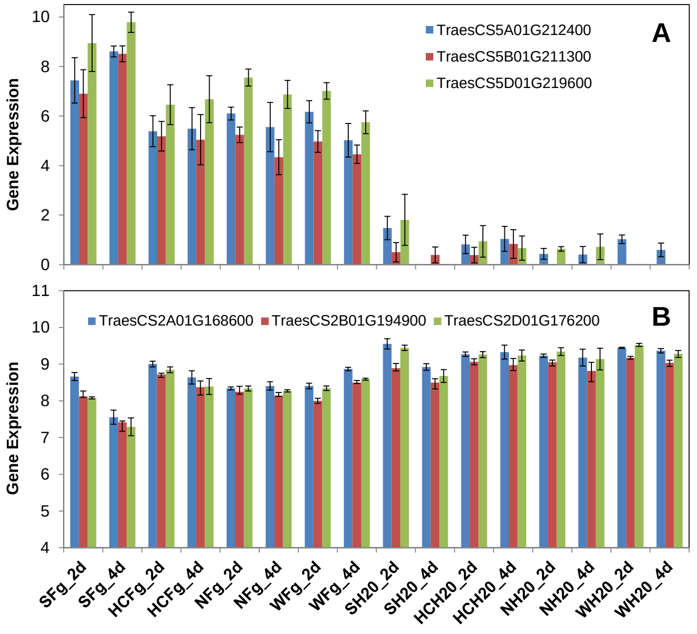

Figure S1. The two groups of genes currently (IWGSC-RefSeqv1.0) annotated as erect panicle 2 protein genes. **A**: the three in the network as key bub genes from Chromosomes 5A, 5B and 5C; **B**: the three not in the network and from Chromosomes 2A, 2B and 2C.
